# Neural Mechanisms Underlying the Processing of Complex Sentences: An fMRI Study

**DOI:** 10.1162/nol_a_00011

**Published:** 2020-06-01

**Authors:** Margreet Vogelzang, Christiane M. Thiel, Stephanie Rosemann, Jochem W. Rieger, Esther Ruigendijk

**Affiliations:** Institute of Dutch Studies, University of Oldenburg, Oldenburg, Germany; Cluster of Excellence “Hearing4all,” University of Oldenburg, Oldenburg, Germany; Cluster of Excellence “Hearing4all,” University of Oldenburg, Oldenburg, Germany; Biological Psychology, Department of Psychology, Department for Medicine and Health Sciences, University of Oldenburg, Oldenburg, Germany; Cluster of Excellence “Hearing4all,” University of Oldenburg, Oldenburg, Germany; Biological Psychology, Department of Psychology, Department for Medicine and Health Sciences, University of Oldenburg, Oldenburg, Germany; Cluster of Excellence “Hearing4all,” University of Oldenburg, Oldenburg, Germany; Applied Neurocognitive Psychology, Department of Psychology, University of Oldenburg, Oldenburg, Germany; Institute of Dutch Studies, University of Oldenburg, Oldenburg, Germany; Cluster of Excellence “Hearing4all,” University of Oldenburg, Oldenburg, Germany

**Keywords:** complex sentences, syntactic processing, word order, working memory, cognitive load, fMRI

## Abstract

Previous research has shown effects of syntactic complexity on sentence processing. In linguistics, syntactic complexity (caused by different word orders) is traditionally explained by distinct linguistic operations. This study investigates whether different complex word orders indeed result in distinct patterns of neural activity, as would be expected when distinct linguistic operations are applied. Twenty-two older adults performed an auditory sentence processing paradigm in German with and without increased cognitive load. The results show that without increased cognitive load, complex sentences show distinct activation patterns compared with less complex, canonical sentences: complex object-initial sentences show increased activity in the left inferior frontal and temporal regions, whereas complex adjunct-initial sentences show increased activity in occipital and right superior frontal regions. Increased cognitive load seems to affect the processing of different sentence structures differently, increasing neural activity for canonical sentences, but leaving complex sentences relatively unaffected. We discuss these results in the context of the idea that linguistic operations required for processing sentence structures with higher levels of complexity involve distinct brain operations.

## INTRODUCTION

It is well known in psycholinguistic research that some sentences are more difficult to process than others (e.g., Bader & Meng, 1999; Bahlmann, Rodriguez-Fornells, Rotte, & Münte, 2007; Tun, Benichov, & Wingfield, 2010). Less is known, however, about the possibly differential effects on processing of sentences with different syntactic complexities. To investigate this, we present an fMRI experiment that assesses how listeners process sentences with two different types of syntactic complexity at two levels of cognitive load and assess the brain mechanisms underlying their processing.

The syntactic complexity of sentences can be varied through variations in word order. For German and English, standard (i.e., canonical) word order in a main clause is Subject (S)-Verb (V)-Object (O; see, e.g., Zwart, 1997 for German). However, speakers frequently use structures that deviate from canonical word order for, among other things, pragmatic reasons. As an example, compare sentences (1a) and (1b). Sentence (1a) shows the canonical word order of SVO. In sentence (1b), however, the object is placed before the subject, creating a more complex syntactic structure.

(1)  a. The father is washing the boy.
  b. It is the boy that the father is washing.
(2)  a. Der Igel berührt den Hasen.
*The_NOM_ hedgehog touches the_ACC_ hare.*
  b. Den Hasen berührt der Igel.
*The_ACC_ hare touches the_NOM_ hedgehog.*

German has relatively rich morphology, which enables relatively free word order. For example, it allows for object-initial sentences, in which the arguments switch places compared to subject-initial sentences while keeping the lexical material constant (compare the SVO sentence in [2a] and the OVS sentence in [2b]). Such sentences have the same meaning in the sense of who did what to whom, but differ pragmatically, for instance (2b) could occur in a story with several animals, in which the hedgehog is touching some but not other animals, and one would like to stress the importance of the hare as the one being touched, and not some other animal. (2a) would then be the version used where the animals are doing several things to each other (e.g., pushing, kissing, touching), and one wants to describe the touching event: the hedgehog is touching the hare. Importantly, both word orders are used in German.

In linguistic theory, it is assumed that different word orders are derived through distinct linguistic operations (e.g., Chomsky, 1981). This linguistic theory makes no explicit claims about processing or the neural mechanisms underlying processing. However, if we assume that different word orders are derived through distinct linguistic operations, differences in the processing of different word orders would also be expected. More specifically, it has been argued that different linguistic operations would be reflected in distinct (neural) processing of sentences with different word orders (Shetreet & Friedmann, 2014). We will refer to this notion as the *linguistic operations* account from now on.

When comparing structurally complex sentences with noncanonical word order to sentences with canonical word order, it is known that complex sentences are generally more difficult to process (e.g., Bader & Meng, 1999; Bahlmann et al., 2007 [embedded clauses]; Carroll & Ruigendijk, 2013 [OVS sentences]; Friederici, Fiebach, Schlesewsky, Bornkessel, & von Cramon, 2006; Tun et al., 2010 [object relative clauses]). For example, object-initial sentences in German have been found to elicit longer reading times (Hemforth, 1993) and more interpretation errors (mistakes in identifying who did what to whom; see, e.g., Carroll, Uslar, Brand, & Ruigendijk, 2016) compared to subject-initial sentences. Such effects of complex sentences compared to canonical sentences have been studied extensively in the literature, but less attention has been given to investigating the processing of different types of noncanonical, complex sentences, which are of particular interest when evaluating the distinct linguistic analyses asserted by the linguistic operations account.

Auditory processing of complex sentences has consistently been found to increase neural activity compared to processing of less complex sentences in the literature, which consists mainly of studies on English, German, and Hebrew. Several brain regions seem to be involved in the processing of multiple types of complexity, or even of complex sentences in general, such as Broca’s area in the inferior frontal gyrus (e.g., Ben-Shachar, Hendler, Kahn, Ben-Bashat, & Grodzinsky, 2003; Ben-Shachar, Palti, & Grodzinsky, 2004; Caplan, Alpert, & Waters, 1999; Röder, Stock, Neville, Bien, & Rösler, 2002; Santi & Grodzinsky, 2010; Shetreet, Friedmann, & Hadar, 2009; Stromswold, Caplan, Alpert, & Rauch, 1996; even independent of modality, Bahlmann et al., 2007; Bornkessel, Zysset, Friederici, von Cramon, & Schlesewsky, 2005; Constable et al., 2004; Friederici et al., 2006; Grewe et al., 2005; Just, Carpenter, Keller, Eddy, & Thulborn, 1996) and posterior superior temporal regions, including Wernicke’s area (Ben-Shachar et al., 2003, 2004; Constable et al., 2004; Just et al., 1996; Santi & Grodzinsky, 2010), which are traditionally considered as auditory and language processing areas. Notably, Friederici et al. (2006) found, in a reading experiment with increasing numbers of argument permutations, that activity in the left inferior frontal gyrus (IFG) increases as a function of syntactic complexity, and similarly Makuuchi, Grodzinsky, Amunts, Santi, and Friederici (2013) found that, in reading, increasing movement distance (as a manipulation of a linguistic operation) increases activity in the same region (although not linearly). In a recent meta-analysis of 54 fMRI studies, Rodd, Vitello, Woollams, and Adank (2015) confirmed the critical role of the posterior left IFG and posterior temporal regions in different types of syntactic processing. Investigating different noncanonical word orders, specifically two types of linguistic operations, verb movement and wh- (who, what, where, when, why) movement, Shetreet and Friedmann (2014) found that in Hebrew, OSV compared to canonical SVO sentences (wh- movement) increased activity in the left IFG and bilateral posterior temporal regions, whereas VSO (verb movement) compared to canonical sentences increased activity in the left inferior occipital gyrus. When comparing OSV sentences directly to VSO sentences, they found increased activity in left inferior frontal and temporal regions and in medial superior frontal regions. Shetreet and Friedmann (2014) conclude that processing different word orders activates different brain areas, reflecting distinct underlying linguistic operations (wh- movement vs. verb movement). Thus, some regions’ involvement may be more specific for the type of complexity, such as inferior occipital regions for processing sentences in which the verb precedes its arguments (Shetreet & Friedmann, 2014), the middle frontal gyrus for processing object-initial sentences (Röder et al., 2002), or the superior frontal gyrus for producing verb-second clauses (in Dutch, Den Ouden, Hoogduin, Stowe, & Bastiaanse, 2008). In addition, there is a large body of research on (temporarily) ambiguous sentences (e.g., Bahlmann et al., 2007; Bornkessel et al., 2005; Friederici, Mecklinger, Spencer, Steinhauer, & Donchin, 2001; Friederici, Steinhauer, Mecklinger, & Meyer, 1998), but since we are interested in the processing of complexity rather than garden paths or ambiguity resolution, these will not be reviewed here.

To sum up, previous research has shown effects of complexity, that is, effects of processing a sentence with noncanonical word order. In addition, we know that in linguistics different word orders are explained by different linguistic operations (e.g., Chomsky, 1981). It is still largely unknown, however, whether these different linguistic operations indeed result in distinct patterns of neural activity. In the current study, we acoustically presented sentences with two types of linguistic complexity, that all contain the same syntactic relation of who did what to whom, to compare their neural processing. We used several cognitive measurements as well as a dual-task paradigm to examine the role of cognitive capacities and cognitive load in the processing of complex sentences. Specifically, a visual change-detection task was used to increase demands of visual attention and executive control (which we will refer to with the general term of cognitive load) without taxing working memory. Importantly, this visual task should not interfere with the auditory processing, but may interact with the cognitive control required to process the different word orders. This would be in line with previous findings linking cognitive control explicitly to syntax in comprehension (in children, Engel de Abreu, Gathercole, & Martin, 2011) and to the syntactic processing of sentences in general (Novick, Trueswell, & Thompson-Schill, 2005). If the processing of different word orders can indeed be explained by distinct linguistic operations, then increased cognitive load may influence the processing of sentences with different word orders in different ways. If, on the other hand, the processing of different word orders relies purely on differences in processing load rather than linguistic operations and analyses, then increased cognitive load would be expected to influence the processing of sentences with different word orders in similar ways. More specifically, memory-based accounts argue that processing cost is the result of the distance between a moved element and its original position, regardless of the type of movement, in the sense that the larger the distance, the more processing costs, which could be reflected in stronger activation patterns (e.g., Grodner & Gibson, 2005; Lewis, Vasishth, & Van Dyke, 2006). Although admittedly this is a somewhat simplified description of these accounts, which additionally take into account other influences such as feature overlap, and few claims have been made about neural mechanisms underlying sentence processing based on these accounts, a memory-based explanation may be expected to result in qualitatively similar activation patterns for different linguistic operations, that could differ in strength. Experience-based accounts (e.g., Hale, 2001; Levy, 2008), in turn, assume that processing is affected by the frequency of occurrence of a certain pattern. Crucially, this would, in our interpretation, also not result in qualitatively different activation patterns for different word orders, but in differences in the amount of activation (see Shetreet & Friedmann, 2014, for a further discussion of these predictions).

Here we investigated these predictions with older adults, who are expected to show more difficulties with complex sentence processing (cf. e.g., Emery, 1985; Kemper, 1992; Wingfield, McCoy, Peelle, Tun, & Cox, 2006) and a wider spread in their cognitive capacities than younger adults; this would create the opportunity to better investigate the correlations between cognitive measurements and linguistic performance, as well as the influence of cognitive load. We measured working memory capacity, as it has been argued that more information has to be kept active in working memory in object-initial sentences compared to subject-initial sentences (Schlesewsky, Fanselow, Kliegl, & Krems, 2000). Additionally, measurements of vocabulary and cognitive flexibility were taken, as these have been found to influence older adults’ performance on sentence processing in adverse listening conditions (McAuliffe, Gibson, Kerr, Anderson, & LaShell, 2013; Rosemann et al., 2017).

Our experiment used four word order conditions—canonical sentences, object-initial sentences, adjunct-initial sentences, and adjunct-initial sentences in which the object precedes the subject (see Table 1)—to test the main hypothesis that different noncanonical sentences are processed in distinct ways, which would be in line with our predictions based on the linguistic operations account. These sentence conditions represent the operations of wh-movement (movement of an argument, creating a sentence in which the object precedes the subject) and adjunct movement (fronting of the adjunct, creating a sentence in which the verb precedes its arguments). Note that in German main clauses, the surface order is verb-second for finite verbs. In subordinate clauses, the surface order is verb-final, and there is discussion on which of these is the actual underlying order (e.g., Broekhuis, 2006). There is, however, general agreement in the literature that there is a difference between moving an adjunct and moving an argument over the verb to the first position of the sentence (see, e.g., Cinque, 1990, or Rizzi, 1997). An important difference between the two types of movement is that argument movement changes the theta-role hierarchy (as in, e.g., Jackendoff, 1990) in which the agent should precede patient, whereas adjunct movement affects the position of the verb in relation to its arguments in German. Both of these movements should arguably affect sentence processing. Regarding the specific sentence conditions, we expect both object-initial sentences (in line with Carroll et al., 2016) and adjunct-initial sentences to be more difficult to process and interpret than canonical sentences, and that changing the order of the subject and object results in different processing than changing the position of the adjunct (in line with Shetreet & Friedmann, 2014). Based on the findings of Bahlmann et al. (2007), Bornkessel et al. (2005), Röder et al. (2002), and Shetreet and Friedmann (2014), we expect increased activity in the left IFG and temporal regions for object-initial sentences compared to canonical sentences. Based on the findings of Shetreet and Friedmann (2014), we expect increased activity in occipital regions for adjunct-initial sentences compared to canonical sentences and increased activity in inferior frontal, superior frontal, and temporal regions for object-initial sentences compared to adjunct-initial sentences. The fourth word order condition, with the adjunct at the beginning and the object before the subject (AVOS), is a new addition to the literature, for which we expect combined effects of object-initial and adjunct-initial sentences. Note that these predictions stem from previous literature on the neural processing of these types of complex sentences rather than the linguistic operations account or memory-based accounts directly, as these do not provide any specific predictions about the localization of neural processing of complex sentences. Although the dual task manipulation is more explorative and therefore the hypotheses less precise, we expect increased cognitive load to make all sentences more difficult to interpret correctly and to affect the processing of the different sentences distinctly, which again would be in line with the linguistic operations account. Our reasoning is that if underlying linguistic operations are not the same, they may not be affected to the same degree by a general cognitive load as induced by the dual task. Based on previous research on the influence of cognitive capacities, we expect working memory (Payne et al., 2014; Vos, Gunter, Schriefers, & Friederici, 2001), vocabulary, and cognitive flexibility (McAuliffe et al., 2013; Rosemann et al., 2017) to correlate with the interpretation and processing of complex sentences.

**Table 1.** Example of an experimental item in the four different sentence conditions, each containing a subject (S), verb (V), adjunct (A), and object (O)

**Condition**	**Example sentence**
SVAO	Der Igel berührt am Montag den Hasen.
	*The_NOM_ hedgehog touches on Monday the_ACC_ hare.*
OVAS	Den Hasen berührt am Montag der Igel.
	*The_ACC_ hare touches on Monday the_NOM_ hedgehog.*
AVSO	Am Montag berührt der Igel den Hasen.
	*On Monday touches the_NOM_ hedgehog the_ACC_ hare.*
AVOS	Am Montag berührt den Hasen der Igel.
	*On Monday touches the_ACC_ hare the_NOM_ hedgehog.*

## MATERIALS AND METHODS

### Participants

Twenty-four volunteers participated in the study. One participant had to be excluded due to technical issues and one other participant was excluded due to a lack of responses during the dual task. Twenty-two participants remained for analysis (15 females, aged 51–70, mean age 61; 0, *SD* 6; 3). The participants had normal hearing as tested with an individual pure tone audiogram (PTA-4 ≤ 15 dB, PTA-high ≤ 25 dB) and normal or corrected-to-normal vision. Participants performed the Montreal Cognitive Assessment task (MoCA; Nasreddine et al., 2005), a concise screening tool for mild cognitive impairment, which rendered a mean group score of 27.6 out of 30, indicating normal cognitive functioning. The participants were all native speakers of German and reported no language impairment and no psychiatric or neurological disorders. They were all right-handed. The ethics committee of the University of Oldenburg, Germany, approved the study (approval number Drs. 28/2017) and written informed consent was obtained from all participants. Participants received monetary compensation for their participation.

### Materials and Design

The experiment used acoustically presented German sentences, each followed by two pictures for a picture-selection task. Each sentence consisted of a subject, a transitive verb, a temporal adjunct, and an object. Four different sentence conditions were used: SVAO sentences, which have canonical word order, OVAS sentences in which the object is placed sentence-initially, adjunct-initial AVSO sentences in which the verb is placed before its arguments, and adjunct-initial AVOS sentences in which the subject-object order is additionally manipulated. See Table 1 for an example of each of the four conditions. The subject and object of a sentence were always animate masculine nouns, to allow for unambiguous nominative and accusative markers on the determiners. The temporal adjunct always consisted of “am” (on) followed by a two-syllable day of the week.

Sentences were created based on the OLACS corpus (Uslar et al., 2013): Adjectives were removed from the original sentences and temporal adjuncts were added. An online questionnaire was performed with 165 participants as a pretest to check for the reversibility of the argument roles. Only sentences that were equally plausible with and without role reversal were selected for the main experiment; sentence pairs for which the difference in plausibility score exceeded two standard deviations from the mean were excluded. Forty items remained for the main experiment.

All sentences were recorded by a native German-speaking woman at 44,100 Hz sampling rate. The mean sentence length was 2,653 ms. A high-pass filter of 50 Hz was applied post-recording. The sentences were adjusted to 36.3 RMS and calibrated at 100 dB. Using the Oldenburg (Matrix) Sentence Test (Wagener, Brand, & Kollmeier, 1999a, 1999b; Wagener, Kühnel, & Kollmeier, 1999), the loudness of the stimuli was adjusted for each participant individually to 80% intelligibility during the MRI measurements to ensure that all participants could hear the stimuli equally well. The average adjusted loudness of stimuli presentation was 72 dB (*SD* = 7.0). These were played over MR compatible headphones (Opto Active, Optoacoustics Ltd, Israel) after applying noise cancellation to eliminate part of the scanner noise.

After each sentence, two pictures were displayed. These presented both characters mentioned in the sentence performing the mentioned action. Note that the experiment thus did not test the interpretation of the adjunct. Rather, it tested the influence of the adjunct manipulation, as well as the subject-object order manipulation, on the sentence structure, and therefore on sentence processing, as a whole. Participants could indicate the picture that best fit the sentence with a response box: the left button for the left picture and the right button for the right picture (with the right index and right middle finger, respectively). The location of the target picture on the screen (left or right) was counterbalanced across trials. Visual stimulation was accomplished by a projector (DATAPixx2, VPixx Technologies Inc.) and a screen that was positioned behind the MRI at a distance of 50 cm from eye to screen.

The experiment tested the effect of cognitive load on sentence processing by means of a secondary task. As the secondary task, a fixation cross change detection task was used, which was chosen because it is a visual task that taxes cognitive control mechanisms without interfering with sensory processing in the primary task. In the dual task condition, either the horizontal or vertical line of the fixation cross, which was always displayed on the screen during sentence presentation, could become slightly larger. Importantly, the fixation cross change appeared during sentence presentation. Participants had to pay attention to the fixation cross change and press a button when they detected it; the cross then turned grey until the pictures appeared on the screen. No action was required when no change was detected.

Finally, a baseline condition without sound, but with pictures, was added to check the data recording and analysis. In this condition, participants were instructed to select one of the pictures randomly. The four sentence conditions and one silent baseline, presented either as a single task or in combination with a secondary task (dual task condition), constituted a 5 x 2 within-subjects design. The experiment consisted of 240 trials in total (24 critical trials per condition), distributed over two sessions with a short break in between. In each session, 120 trials were presented, divided into six blocks of 20 trials each. Each block presented either the single or the dual task; these two types of blocks were alternated. Two pseudo-randomized lists were created to prevent potential order effects.

**Figure 1.**
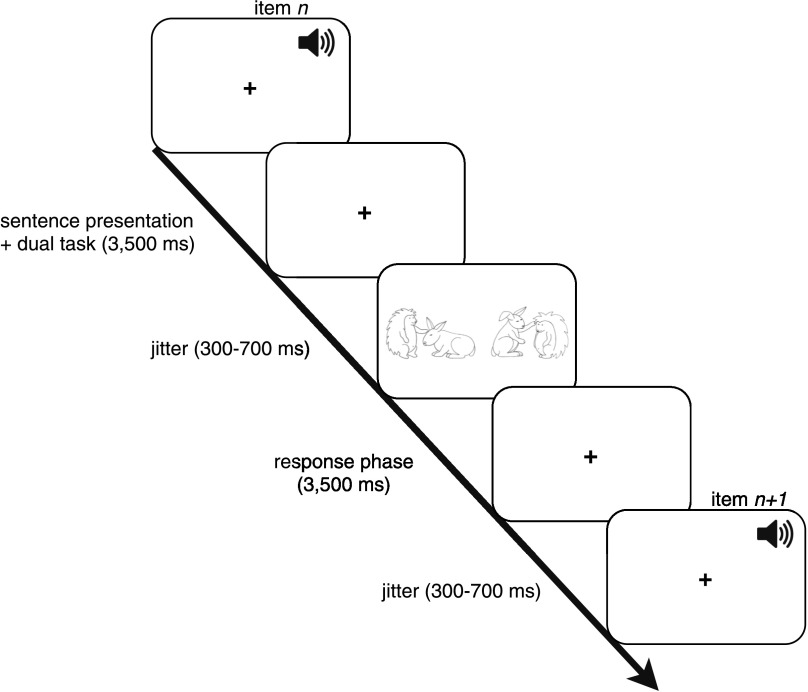
Overview of the presentation of the stimuli within one trial (pictures adjusted from Wendt, Brand, & Kollmeier, 2014; Wendt, Kollmeier, & Brand, 2015).

Each trial lasted 8 s. Within a single trial, the critical sentence (or silent baseline) was presented acoustically accompanied by a fixation cross on the screen, followed by a jitter of 300 to 700 ms, before two pictures appeared on the screen for the response phase. The response phase lasted 3,500 ms. No feedback was given throughout the practice trials and the main experiment. After the response phase, a fixation cross appeared again with a jitter of 300 to 700 ms, before the next trial started automatically. A schematic overview of the presentation of a trial is depicted in Figure 1. Instructions on whether the single or dual task should be performed were displayed for 10 s prior to a new block of trials.

### Cognitive Tests

#### Digit span

A standard backwards digit span task was applied as a measurement of working memory (WM) capacity. Participants heard a sequence of digits (1–9) and were asked to repeat this sequence in reverse order. Increasingly longer sequences were tested, each sequence length twice, starting at length two and ending when the participant was not able to correctly repeat any instances of a sequence length. For each correctly reported sequence, participants received one point. The longest possible sequence was eight digits; therefore, the maximal number of points was 14. The mean score of our participants was 7.0 (*SD* = 2.4).

#### Vocabulary

A German vocabulary test called “Wortschatztest” (Schmidt & Metzler, 1992) was used as a measure of verbal intelligence. Participants were presented with rows of six words in which five nonwords and one existing word were included and were asked to detect the existing German word without guessing. Increasingly difficult words were tested. For each correctly identified word, participants received one point. The task consists of 42 rows of words; therefore, the maximal number of points was 42. The mean score of our participants was 33.6 (*SD* = 2.7).

#### CTMT

The Comprehensive Trail-Making Test (CTMT; Reynolds, 2002) was used as a measure of cognitive flexibility and task switching abilities. It uses a visual search task in which participants have to connect encircled numbers and letters in ascending order on paper. Two CTMT subtests were conducted: trail 1 with numbers from 1 to 25 and trail 5 with numbers and letters that have to be connected in alternation (i.e., 1-A-2-B-3-C…). The time from start until completion of a trail was measured. Any errors made by the participant were corrected immediately. Participants’ final score on the CTMT was calculated as the difference in completion time between trail 1 and trail 5; a lower score thus reflects better cognitive flexibility. The mean score of our participants was 26.4 s (*SD* = 17.8).

### Procedure

Participants were tested individually in a quiet room at the University of Oldenburg. First, audiogram measurements were taken in a soundproof booth. Then, the CTMT, MoCA, and digit span task were conducted, followed by a first practice for the main experiment, before starting the part of the study that took place in the MRI scanner.

The tests in the scanner were split into two parts, with a break in between. The Oldenburg Sentence Test, a second practice for the main experiment, and the first session of the main experiment were performed in the first part. After this first part, participants would have a break outside the scanner, in which they completed the vocabulary task. After a 10- to 15-min break, the second session of the main experiment, a structural scan (T1, longitudinal or spin-lattice relaxation time), resting state measurements, and DTI measurements were done in the scanner. The resting state and DTI data will not be reported in this article. Each session of the main experiment took about 18 min. The complete procedure took around 3 hr.

### Behavioral Data Analysis

All responses to the picture-selection task were coded as either correct or incorrect. Subsequently, the rates of correct responses were calculated for each of the four sentence conditions in both the single and the dual task condition. We examined the differences between the sentence conditions, the effect of the dual task, and the relationship between responses on the linguistic task and scores on the cognitive tests (digit span, vocabulary, and CTMT). Post hoc Bonferroni-corrected pairwise comparisons identified which sentence conditions differed from each other.

The responses were analyzed using binomial generalized linear mixed-effect-models in R. Sentence condition was coded in terms of subject-object order (two levels) and adjunct position (two levels) using effect coding (i.e., −0.5, 0.5). We examined the effects of *subject-object order*, *adjunct position, task condition* (i.e., single vs. dual task) and any interactions (fixed effects) on the rate of correct responses (dependent variable). Note that for the offline comprehension, we only expected an effect of subject-object order and not necessarily of adjunct position, since the task only directly addressed this aspect. To put it differently, for finding the correct picture, the position of the adjunct was irrelevant. Adjunct position was hence only included here for the sake of completeness. A maximal (converging) random effects structure was used, with random intercepts for participants and items and random slopes for subject-object order for subjects and for subject-object order and adjunct position for items. Additional factors such as list, session, and trial order were tested for warranted inclusion in the model; only session improved the model and was therefore included. The influence of digit span, vocabulary, and CTMT was assessed subsequently by testing their warranted inclusion in the model as well as possible interactions with subject-object order and adjunct position; digit span, vocabulary, and CTMT were all included in the model as well as an interaction between subject-object order and digit span.

### MRI Data Acquisition

The functional and anatomical MRI measurements were conducted with a 3T Siemens Magnetom Prisma MRI scanner located at the University of Oldenburg. A 20-channel head coil was used. Echo-planar imaging was used to measure a sequence with blood oxygen level dependent, or BOLD, contrast (repetition time [TR] = 1,800 ms, echo time [TE] = 30 ms, flip angle = 75 degrees, *df* = 20, slice thickness = 3 mm, field of view = 192 cm, 33 slices). For the first part of the main experiment, 600 whole-brain volumes were acquired and for the second part (which included four extra warm-up trials) this number was 617. Additionally, T1-weighted anatomical images were recorded (TR = 2,000 ms, TE = 2.07 ms, flip angle = 9°, slice thickness = 0.75 mm, 224 slices).

### fMRI Data Analysis

Image processing and analysis was performed using SPM12 (Statistical Parametric Mapping, Wellcome Department of Imaging Neuroscience, University College London, http://www.fil.ion.ucl.ac.uk/spm). Preprocessing for each session of the imaging data included motion correction and realignment to the first image for the functional data, coregistration, segmentation, and normalization to the Montreal Neurological Institute, or MNI, space for the functional and anatomical data, and smoothing with a Gaussian filter (8 mm kernel) for the functional data.

For the analysis of the fMRI data, we focused on the time window from the onset of the sentence until the onset of the pictures. Note that this time window is 3,500 ms + jitter, so the time window varied between 3,800 ms and 4,200 ms for every trial. At the request of one of the reviewers, the data were additionally analyzed using a shorter duration (including only the audio, without the jitter or pause following the audio). The results of this additional analysis were qualitatively similar to the analysis presented in this article, so the results from the longer time will be reported in the Results section. One case in which the results from the different analyses differed, namely, activation in the left IFG for AVSO sentences, will be discussed in the Discussion section. First-level analyses were done per participant using a general linear model. We applied a high-pass filter of 128 s and accounted for serial correlations with an Auto-Regressive model of order 1—AR(1). Head movement parameters were added as regressors for each session separately. No participants were excluded due to excessive head movements (>3 mm). The models were used to estimate the regression coefficients for the sentences, with which the contrasts 1) OVAS > SVAO (object-initial compared to canonical word order), 2) AVSO > SVAO (adjunct-initial compared to canonical word order), 3) AVOS > SVAO (adjunct-initial, object-before-subject compared to canonical word order), 4) OVAS > AVSO (object-initial compared to adjunct-initial word order), 5) AVOS > OVAS (adjunct-initial, object-before-subject compared to object-initial word order), and 6) AVOS > AVSO (adjunct-initial, object-before-subject compared to adjunct-initial word order) were calculated for both the single and the dual task. For the group (second-level) analyses, these estimates were taken to calculate the effects of syntactic complexity in the single task and in the dual task with simple *t* tests. Paired *t* tests were used to examine the interaction between syntactic complexity and cognitive load. Effects are reported as significant when exceeding a cluster-level (Family-Wise Error) corrected threshold of *p* < 0.05 (with a *p* < 0.001 cluster-forming threshold). To localize the brain regions and Brodmann areas (BA) we used SPM12, xjView (http://www.alivelearn.net/xjview), and BioImage Suite’s MNI2TAL converter (http://sprout022.sprout.yale.edu/mni2tal/mni2tal.html). Subsequently, conjunction analyses were performed to investigate which regions showed common activation in different contrasts by creating a flexible factorial design with each of the three main contrasts of complex condition > SVAO. Finally, the influence of different cognitive capacities was investigated with multiple regression analyses, specifying the three factors of interest (digit span, vocabulary, CTMT) as covariates.

## RESULTS

### Behavioral Results

**Figure 2.**
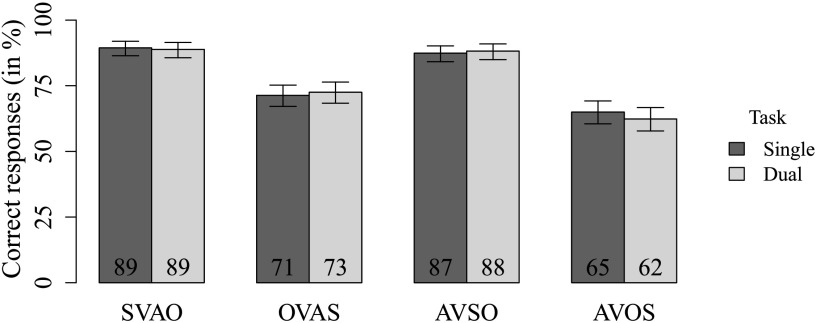
Percentages of correct responses in the linguistic task as a function of sentence type and load. Error bars are 95% CI derived from logistic analysis. S = subject, V = verb, A = adjunct, O = object.

The behavioral data are shown in Figure 2. The results showed main effects of subject-object order (*β* = −3.34; *z* = −6.66; *p* < 0.001) and adjunct position (*β* = −0.42; *z* = −3.69; *p* < 0.001), indicating that subject-before-object sentences were responded to correctly more often than object-before-subject sentences and that adjunct-third sentences were responded to correctly more often than adjunct-initial sentences. Post hoc pairwise comparisons showed that all conditions significantly differed from each other except for the SVAO and AVSO conditions (see Table 2). No effect of task type (single or dual task) was found (*β* = −0.06; *z* = −0.66; *p* = 0.51). No significant interactions were found (all *p*’s > 0.05).

**Table 2.** Pairwise comparisons between the responses to the different sentence conditions in the behavioral task

	***β***	***z*-value**	***p* value**
OVAS − SVAO = 0	−1.16	−9.41	< 0.001
AVSO − SVAO = 0	−0.13	−0.95	0.77
AVOS − SVAO = 0	−1.54	−12.70	< 0.001
AVSO − OVAS = 0	1.03	8.56	< 0.001
AVOS − OVAS = 0	−0.38	−3.89	< 0.001
AVOS − AVSO = 0	−1.41	−11.92	< 0.001

*Note*. S = subject, V = verb, A = adjunct, O = object.

The model furthermore showed main effects of vocabulary and CTMT (respectively, *β* = 0.18; *z* = 3.96; *p* < 0.001 and *β* = −0.02; *z* = −3.05; *p* < 0.01), indicating that participants who performed better on the vocabulary task and CTMT task also performed better on the linguistic task. Finally, an interaction between digit span score and subject-object order (*β* = 0.24; *z* = 3.57; *p* < 0.001) indicates that participants with a better digit span score also performed better on the object-before-subject conditions in the linguistic task. Please see Vogelzang, Thiel, Rosemann, Rieger, and Ruigendijk (2019) for a more detailed description and plots of the effects of cognitive capacities on the offline sentence comprehension.

### Imaging Results

#### Single task

**Figure 3.**
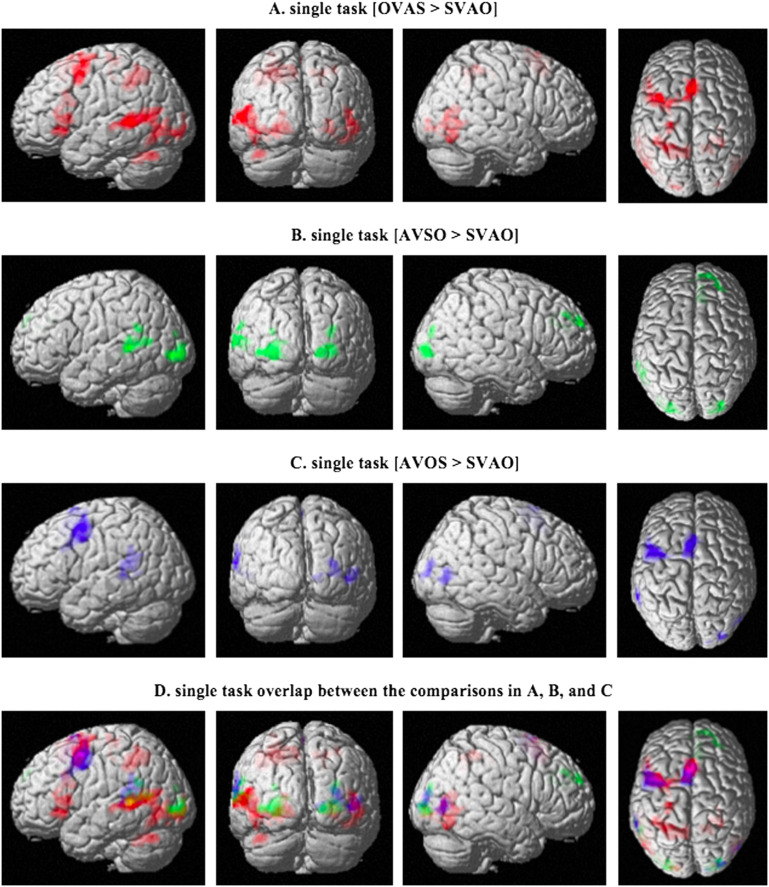
Activation patterns for the comparisons between sentence types in the single task. (A) shows the comparison between object-initial word order and canonical word order (OVAS > SVAO), (B) shows the comparison between adjunct-initial word order and canonical word order (AVSO > SVAO), and (C) shows the comparison between AVOS and the canonical SVAO. Activated areas include the left auditory cortex and Broca’s area in (A), left auditory cortex and superior frontal regions in (B), and left auditory cortex and middle frontal regions in (C), *p* < 0.05; family-wise error corrected on the cluster level. The overlap between these comparisons is shown in (D). S = subject, V = verb, A = adjunct, O = object.

**Figure 4.**
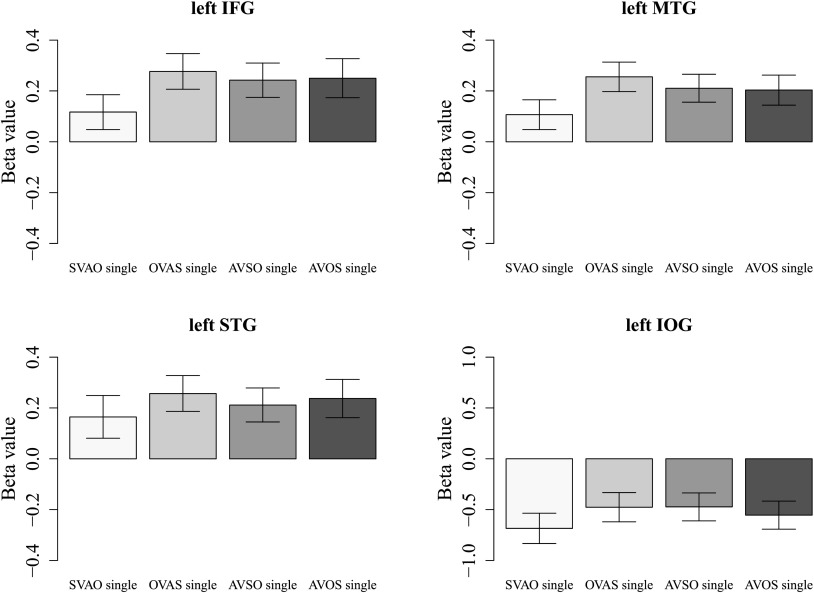
Average beta values extracted from the inferior frontal gyrus (IFG), middle temporal gyrus (MTG), superior temporal gyrus (STG), and inferior occipital gyrus (IOG) in all four sentence conditions in the single task. Beta values were extracted from the entire mentioned regions using WFU PickAtlas (Maldjian, Laurienti, & Burdette, 2004; Maldjian, Laurienti, Kraft, Burdette, & Kraft, 2003). Error bars indicate standard error of the mean. S = subject, V = verb, A = adjunct, O = object.

A first analysis investigated the effects of sentence complexity in the single task. The first comparison, testing the effect of object- vs. subject-initial word order (OVAS > SVAO; Figure 3A, Table 3), showed activity in left cerebellar and temporal areas (BA 22), left inferior frontal areas (including Broca’s area, BA 45 and BA 47), and superior parietal areas. Additional activity was found in the inferior occipital gyrus and the left postcentral gyrus. The second comparison, testing the effect of adjunct-initialization (AVSO > SVAO; Figure 3B, Table 3), showed activity in occipital areas and the left middle temporal gyrus (MTG; BA 21/22). Additional activity was found in right superior frontal areas. The third comparison, testing the effect of adjunct-initial word order with the object before the subject vs. canonical word order (AVOS > SVAO; Figure 3C, Table 3), showed activity in the left middle frontal gyrus (MFG; BA6) and left superior temporal gyrus (STG), with additional activity in the left supplementary motor cortex and right superior occipital gyrus. The overlap between these three comparisons is shown in Figure 3D. Finally, we compared the noncanonical, complex conditions to each other (OVAS > AVSO, AVOS > OVAS, and AVOS > AVSO). Although the comparisons of these complex sentences to the canonical SVAO word order showed different activation patterns, the differences in activity between the complex sentences did not reach significance. To further examine the activity in each sentence condition, we extracted the beta values for these conditions in four key regions for which hypotheses were formulated (the left IFG, left middle and superior temporal gyri, and left inferior occipital gyrus) for visual inspection. These plots, shown in Figure 4, confirm the overall differences in activity in these regions between complex sentence conditions on the one hand and the canonical SVAO condition on the other. The key regions show little differences in activity between the noncanonical sentences, which is in line with the whole-brain–level analyses, in which differences in activity between the complex sentences did not reach significance. One explanation for this may lie in the large variation between participants, which is exemplified by their individual activation patterns in the contrast of complex sentences (all three types) vs. canonical SVAO sentences in the Supporting Information file 1.

**Table 3.** MNI coordinates for the different comparisons under investigation in the single task

**Comparison**	**Peak coordinates (x, y, z)**	***Z*-value**	**Cluster size**	**Brain region**
single [OVAS > SVAO]	(−40, −68, −28)	5.09	2,765	Left cerebellum exterior
	(−48, 24, −4)	4.64	2,311	Left frontal operculum
	(−32, −50, 50)	4.59	1,445	Left superior parietal lobule
	(52, −70, −8)	4.10	843	Right inferior occipital gyrus
	(30, −42, 54)	4.09	549	Right superior parietal lobule
	(−26, −30, 64)	3.99	253	Left postcentral gyrus
single [AVSO > SVAO]	(−24, −92, 2)	5.62	569	Left inferior occipital gyrus
	(−54, −42, 4)	4.28	471	Left middle temporal gyrus
	(18, −94, −2)	4.25	469	Right inferior occipital gyrus
	(12, 30, 28)	4.10	241	Right superior frontal gyrus (medial)
	(8, 60, 30)	3.93	212	Right superior frontal gyrus
single [AVOS > SVAO]	(−46, 4, 54)	5.20	817	Left middle frontal gyrus
	(−6, 14, 56)	4.62	991	Left supplementary motor cortex
	(−52, −38, 4)	4.13	391	Left superior temporal gyrus
	(32, −92, 14)	3.94	244	Right superior occipital gyrus

*Note*. S = subject, V = verb, A = adjunct, O = object.

**Figure 5.**
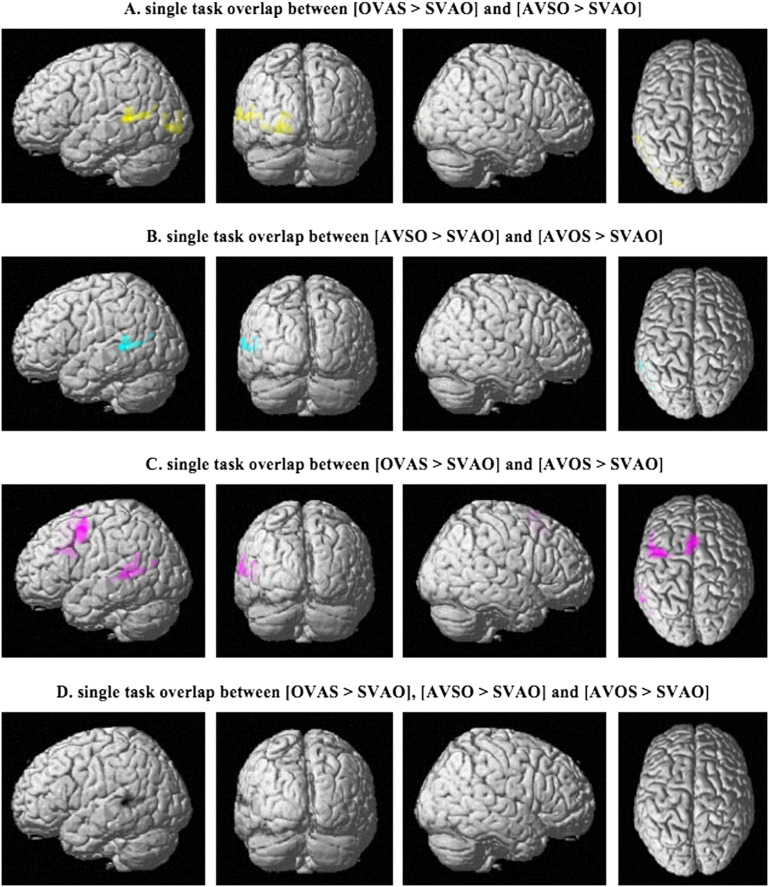
Pairwise overlap in activity between the different sentence conditions in the single task based on conjunction analyses (*p* < 0.05; family-wise error corrected on the cluster level). The overlap between all three comparisons is shown in (D). S = subject, V = verb, A = adjunct, O = object.

Conjunction analyses were used to quantify the overlap between the different comparisons as shown in Figure 3D. These were performed for each set of contrasts separately in the single task (i.e., the overlap between OVAS > SVAO and AVSO > SVAO, the overlap between AVSO > SVAO and AVOS > SVAO, and the overlap between OVAS > SVAO and AVOS > SVAO), as well as the overlap between all three different contrasts. The results, presented in Figure 5 and Table 4, show that although the complex sentence conditions show overlap in their activation patterns, the only region that is activated in all three contrasts is the left superior temporal gyrus (Figure 5D).

**Table 4.** Peak MNI coordinates for the overlap between the different sentence comparisons in the single task (based on conjunction analyses)

**Conjunction**	**Peak coordinates (x, y, z)**	***Z*-value**	**Cluster size**	**Brain region**
[OVAS > SVAO] and [AVSO > SVAO]	(−50, −40, 4)	4.11	422	Left superior temporal gyrus
	(−22, 94, −4)	4.11	439	Left inferior occipital gyrus
[AVSO > SVAO] and [AVOS > SVAO]	(−50, −40, 4)	4.43	349	Left superior temporal gyrus
[OVAS > SVAO] and [AVOS > SVAO]	(−46, 4, 54)	5.65	837	Left middle frontal gyrus
	(−52, −38, 4)	4.89	713	Left superior temporal gyrus
	(−4, 18, 52)	4.73	874	Left supplementary motor cortex
[OVAS > SVAO], [AVSO > SVAO] and [AVOS > SVAO]	(−50, −40, 4)	4.11	348	Left superior temporal gyrus

*Note*. S = subject, V = verb, A = adjunct, O = object.

#### Dual task

The dual task analyses investigated the same contrasts as the single task analyses, as well as the difference between the single and the dual task in these contrasts. Within the dual task, no effects of object-initial word order compared to canonical word order (OVAS > SVAO), of adjunct-initial word order compared to canonical word order (AVSO > SVAO), or of adjunct-initial object-before-subject word order compared to canonical word order (AVOS > SVAO) were found. So, the dual task seems to reduce or even eliminate the effects of syntactic complexity that were found in the single task. When comparing the object-initial to the adjunct-initial condition (OVAS > AVSO), activity was found in the brain stem [peak coordinate (−6, −40, −24), *Z* = 4.14, cluster size = 194].

**Figure 6.**
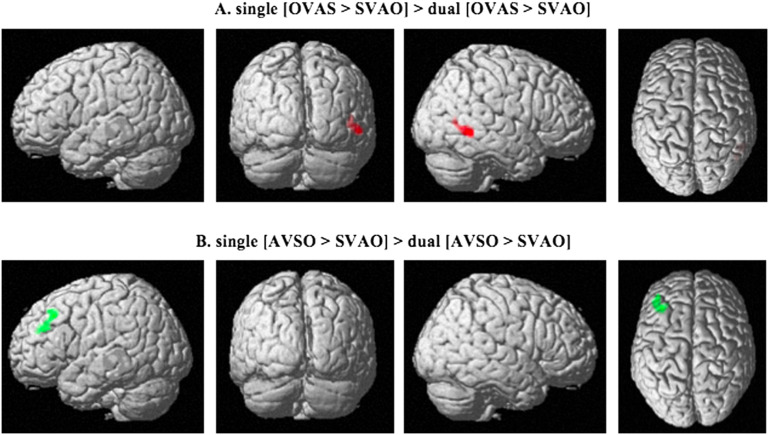
Activation patterns for the comparisons between sentence types in the single vs. dual task. The top row shows the comparison between object-initial word order and canonical word order in the single compared to the dual task (single [OVAS > SVAO] > dual [OVAS > SVAO]). The bottom row shows the comparison between adjunct-initial word order and canonical word order in the single compared to the dual task (single [AVSO > SVAO] > dual [AVSO > SVAO]). Activated areas include the right middle temporal gyrus in (A), and left middle frontal gyrus in (B), *p* < 0.05; family-wise error corrected on the cluster level. S = subject, V = verb, A = adjunct, O = object.

Examining the difference between the single and the dual task, the results show effects for both [OVAS > SVAO] and [AVSO > SVAO] in the [single-dual] comparison, but not in the [dual-single] comparison. Specifically, an increased difference in activity was found in the single task compared to the dual task in the [OVAS > SVAO] contrast in the right MTG (Figure 6A, Table 5) and in the [AVSO > SVAO] contrast in the left MFG (Figure 6B, Table 5). No effects of [AVOS > SVAO], [OVAS > AVSO], [AVOS > OVAS], or [AVOS > AVSO] were found in the [single-dual] comparison or in the [dual-single] comparison. Thus, as in the single task, the dual task comparisons show no differences in the activity between noncanonical conditions.

**Table 5.** Peak MNI coordinates for the different comparisons between the single and the dual task

**Comparison**	**Peak coordinates (x, y, z)**	***Z*-value**	**Cluster size**	**Brain region**
single [OVAS > SVAO] > dual [OVAS > SVAO]	(54, −50, −4)	4.56	238	Right middle temporal gyrus
single [AVSO > SVAO] > dual [AVSO > SVAO]	(−42, 42, 26)	3.88	214	Left middle frontal gyrus

*Note*. S = subject, V = verb, A = adjunct, O = object.

**Figure 7.**
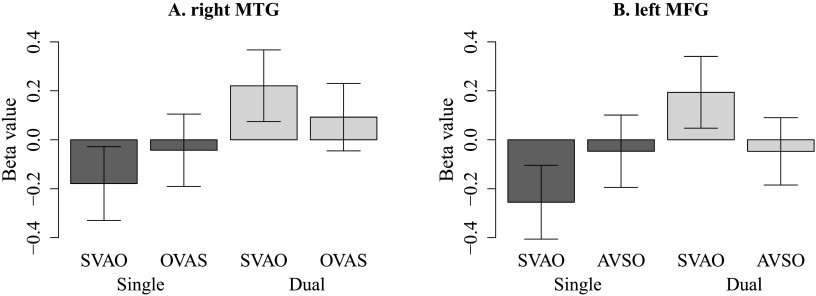
Average beta values extracted from the right middle temporal gyrus (MTG) for the OVAS and SVAO conditions in the single and the dual task (left graph) and from the left middle frontal gyrus (MFG) for the AVSO and SVAO conditions in the single and the dual task (right graph), at the peak values listed in Table 5. Error bars indicate standard error of the mean. S = subject, V = verb, A = adjunct, O = object.

To investigate these effects in more detail, we extracted the beta values from the peak coordinates listed in Table 5 and plotted these for visual inspection. In the right MTG, the OVAS condition is largely unaffected by the task factor, whereas the activity associated with the processing of canonical SVAO sentences is increased in the dual task compared to the single task (Figure 7A). A similar pattern can be seen for the AVSO (unaffected by task) compared to SVAO (affected by task) sentences in left MFG (Figure 7B). Thus, it seems that the processing of canonical SVAO sentences is affected by the dual task more than the processing of complex OVAS or AVSO sentences.

**Figure 8.**
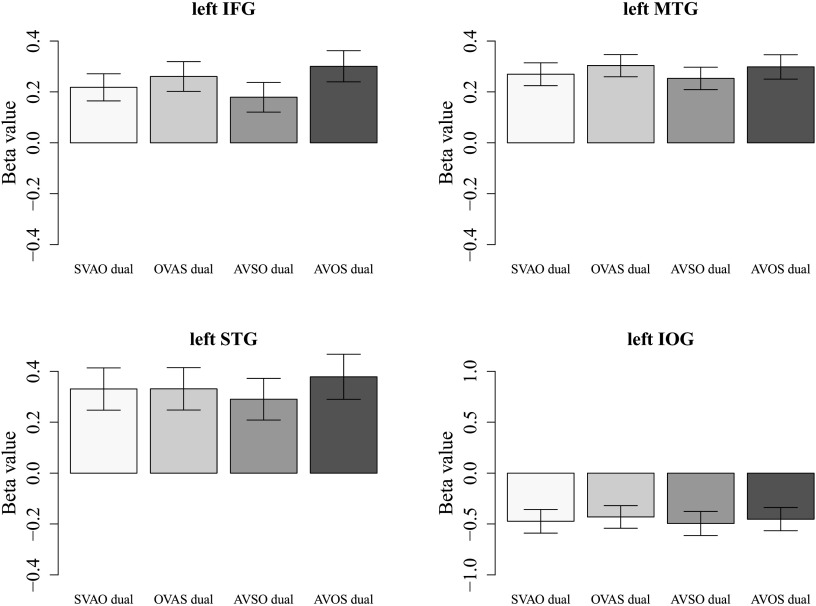
Average beta values extracted from the inferior frontal gyrus (IFG), middle temporal gyrus (MTG), superior temporal gyrus (STG), and inferior occipital gyrus (IOG) in all four sentence conditions in the dual task. Beta values were extracted from the entire mentioned regions using WFU PickAtlas (Maldjian et al., 2003, 2004). Error bars indicate standard error of the mean. S = subject, V = verb, A = adjunct, O = object.

Again, the activity in each sentence condition was further examined by extracting the beta values in the four key regions for visual inspection. The results for the single as well as the dual task conditions are plotted in Figure 4 (single task) and Figure 8 (dual task). The graphs show, in line with previous whole-brain analyses, that whereas in the single task condition SVAO sentences show lower beta values than the complex sentences, in the dual task conditions these differences have decreased, showing little difference in activity between canonical and complex sentence conditions. We statistically investigated these differences with region of interest (ROI) analyses and found a significant effect for the comparison between object-initial word order and canonical word order in the single compared to the dual task (single [OVAS > SVAO] > dual [OVAS > SVAO]) in the left MTG [peak coordinate (−52, −66, 16), *Z* = 3.56, cluster size = 83].

#### Correlations with cognitive capacities

Finally, we investigated the influence of different cognitive capacities with multiple regression analyses for those comparisons that showed significant effects of word order in the previous analyses. Therefore, we examined the relation between the processing of syntactically complex sentences (OVAS > SVAO, AVSO > SVAO, and AVOS > SVAO) in the single task on the one hand and digit span score, vocabulary score, and CTMT score on the other. No correlations between these cognitive capacities and processing of complex sentences were found.

## DISCUSSION

In this study, we investigated the mechanisms underlying the processing of different noncanonical, complex sentences by older adults. Our main hypothesis was that distinct linguistic operations underlie the processing of different noncanonical sentences, which were predicted to be reflected in distinct patterns of neural activity. The behavioral results showed that object-initial sentences and adjunct-initial sentences with the object before the subject lead to different performance levels compared to canonical sentences and compared to each other in the picture-selection task. These results support the idea that these noncanonical word orders affect processing and therefore offline interpretation, even if the interpretation (of the adjunct) is not directly assessed in the task. Adjunct-initial sentences with the subject before the object were not found to be interpreted differently from canonical sentences.

In the imaging data, used to examine the online processing and neural patterns underlying this processing, the results are less clear. Despite the finding that the differences in activity between object-initial and adjunct-initial sentences were not significant, our noncanonical sentence conditions did show clearly different neural processing when compared to canonical sentences. Specifically, object-initial sentences showed activity in the left IFG and temporal regions, which are more frequently associated with object-initialization (Bahlmann et al., 2007; Bornkessel et al., 2005; Röder et al., 2002; Shetreet & Friedmann, 2014) and complex sentence processing in general (Rodd et al., 2015). Adjunct-initial sentences with the subject before the object showed activity in occipital regions, in line with Shetreet and Friedmann (2014), and additionally showed activity in right superior frontal areas. Although typically associated with visual processing, occipital regions have been associated with the processing of complex sentences before (Peelle, McMillan, Moore, Grossman, & Wingfield, 2004; Shetreet & Friedmann, 2014) and are connected to the inferior frontal cortex through a ventral pathway (Friederici & Gierhan, 2013). In congenitally blind adults, occipital regions have been found to be sensitive to syntactic complexity, but not to complexity in mathematical equations (Lane, Kanjlia, Omaki, & Bedny, 2015), and show behavior similar to classic language regions (left frontal and temporal regions) during sentence processing (Bedny, Pascual-Leone, Dodell-Feder, Fedorenko, & Saxe, 2011), although their sighted peers did not display such occipital activity. This indicates that the role of occipital activation in sentence processing requires further research. Finally, adjunct-initial sentences with the object before the subject compared to canonical sentences showed activity that partially overlapped with activity found for the object-initial sentences (left MFG, left STG, and left supplementary motor cortex) and adjunct-initial sentences with the subject before the object (left STG). Interestingly, neither our study nor the study of Shetreet and Friedmann (2014) found increased activity in the IFG for adjunct-initial sentences in which the verb precedes its arguments, whereas many previous studies, which used various sentence structures, systematically found activity in this region (see Rodd et al., 2015, for an overview). It has been shown, however, that effects and activity can vary between different types of sentence manipulations, such as embedding (Santi & Grodzinsky, 2010) and scrambling (Makuuchi et al., 2013) compared to movement or even between different types of movement (Shetreet & Friedmann, 2014), which may explain the absence of an effect in the IFG for our adjunct-initial manipulations. Moreover, in the additional analyses including only the audio (without the jitter or pause following the audio) described in the Materials and Methods section, left IFG activation *did* show for adjunct-initial AVSO sentences, indicating that the length of the time window affects the detection of this activity. Conjunction analyses showed that all noncanonical sentence conditions shared activation in the left posterior STG, which is traditionally considered to be involved in speech and language comprehension, and more specifically in the processing of noncanonical sentences (Friederici, Kotz, Scott, & Obleser, 2010). Thus, we found effects of object-initialization and adjunct-initialization (and thus verb-argument order) that are in line with the literature, and novel results for adjunct-initial sentences with the subject before the object. However, we could not replicate Shetreet and Friedmann’s (2014) findings on the differences in neural activity between noncanonical object-initial and adjunct-initial sentences in our sample of older, German-speaking adults. This may be explained by differences in the experimental design (i.e., our pseudo-randomized design vs. the blocked design that Shetreet and Friedmann used). In addition, the difference in findings may be explained by differences between the two tested languages, such as differences in the case marking system (only the accusative is morphologically marked in Hebrew, through an extra accusative marker “et,” whereas German marks all cases, in our study on the article of the noun phrase). Furthermore, the position of the verb is different in Hebrew and German, which may be an underlying factor. For Hebrew, it has been argued to always be in second position (SVO); whereas for German, it has been argued to be in final position in the underlying word order (see Introduction). Hence, one could argue that in our German sentences, verb movement took place in all four conditions, whereas in Hebrew, verb movement was the actual manipulation in one of the conditions. The finding that there are no significant differences in the neural processing of different complex sentences seems more in line with a memory-based account of sentence processing than a linguistic operations account. However, since absence of evidence is not evidence of absence, these results are to be interpreted with caution.

To investigate the processing of different noncanonical sentences further, a dual task paradigm was used. Contrary to our expectations, the behavioral results showed no differences between the single and the dual task. As the dual task occurred during sentence processing, it is possible that the effects of the increased load were not present anymore during the response phase. In contrast to the behavioral results, the imaging results showed that neural processing in the dual task was different compared to the single task. Specifically, whereas the single task showed differences in activity between canonical sentences on the one hand and noncanonical sentences on the other, these differences disappeared in the dual task. So, increased cognitive load seems to be able to affect the processing of different sentence structures differently, increasing neural activity for canonical sentences, but leaving the noncanonical sentences relatively unaffected. Differences in differential activity for canonical vs. noncanonical sentences between the single and the dual task were found in the right MTG and left MFG. Although these were not the regions that we hypothesized finding differences in, thus making the effects more difficult to interpret, additional examinations of the data suggest similar effects in the key ROIs. In fact, ROI analyses confirmed a similar effect in the left MTG. It is difficult to find direct support for either the linguistic operations account or memory-based accounts in these findings. The findings could be interpreted within the linguistic operations account, which assumes that different word orders are derived from distinct linguistic analyses, which could thus be affected differently by increased cognitive load. However, based on this account we would have expected to find differences between noncanonical sentences as well, which were not found in the data. It is also difficult to reconcile with experience- or memory-based accounts (Hale, 2001; Levy, 2008; Lewis et al., 2006), as in our understanding these accounts would predict all sentences to be affected in a similar way, with the differences between sentences still being present. Moreover, the accounts themselves do not make claims about the neural processing of different sentences, so more precise predictions on the basis of these accounts should be formulated in future work. The finding that different sentence structures are differentially affected by cognitive load may point more toward the linguistic operations account, but certainly there is a role of memory and other cognitive resources in (complex) sentence processing as well; the existence of examples in which different sentences are affected by increased cognitive load in different ways merely suggests that a complete account cannot be solely memory-based. Overall, we tentatively interpret the results as providing support for the idea that distinct mechanisms, that could be explained by distinct underlying linguistic operations, underlie the processing of different sentences. Nevertheless, the nature of these processing differences and the relation between the identified brain regions and underlying processing mechanisms for different sentences remains to be investigated further.

Finally, we examined the influence of several cognitive capacities on sentence processing. In the behavioral data, effects of WM capacity, verbal intelligence, and cognitive flexibility were found, indicating that these cognitive capacities interact with the interpretation of (complex) sentences. In the imaging data, no effects of cognitive capacities were found. The differences between the behavioral and the imaging results could be due to (1) statistical corrections (i.e., at the whole-brain level) for the imaging data, (2) the imaging data testing the correlation of cognitive capacities with differential activity in the different sentence conditions rather than with performance on one condition (as was done for the behavioral data), or (3) individual variation in the brain areas that are influenced by these cognitive capacities. Further research is needed to investigate these different explanations.

Some notes can be made with regards to the experimental design. We chose to test older participants, as these were expected to show more difficulties with the processing of complex sentences (cf. Emery, 1985; Kemper, 1992; Wingfield et al., 2006) and a wider spread in their cognitive capacities than younger adults, which would aid the planned correlational analyses. Indeed, the behavioral results show that our participants had quite some difficulties correctly interpreting the two object-before-subject conditions. This may in part be due to their age and in part be due to the MRI noise (essentially creating a speech-in-noise task), which can influence the processing of syntactically complex sentences (Carroll & Ruigendijk, 2013). However, the neuroimaging analyses did not show the expected effects between different noncanonical sentences and no correlations with cognitive capacities. These comparisons may have been constrained by the limited number of participants (although we had the same number of participants as Shetreet & Friedmann, 2014, namely, 22) and/or the strict corrections that were applied at the whole-brain level (as recommended for SPM12). Future research should therefore try to further clarify the differences in the processing of complex sentences and the cognitive capacities that play a role in this processing, potentially focusing on different populations (e.g., older vs. younger) and different cognitive skill levels (e.g., higher vs. lower WM skills). In addition, sentences with different word orders inevitably also differ pragmatically. Although we acknowledge this confound, previous studies on word order variations have found very similar results for the syntactic processing of canonical compared to noncanonical sentences, and the observed activated areas are therefore assumed to be due to syntactic differences. Finally, as different sentence structures are affected differently and canonical sentences behave more like complex sentences under increased cognitive load, our findings indicate that investigations of language processing under challenging conditions will be particularly relevant, for instance, in hearing-impaired populations or during speech processing in background noise (cf. Carroll & Ruigendijk, 2013).

In conclusion, we presented behavioral and imaging data on the processing of two types of sentence complexity in German. The results are mixed, with the imaging results not replicating differences between the processing of different sentences that were found in previous research (Shetreet & Friedmann, 2014), but at the same time showing differential effects of increased cognitive load for different sentence types. We tentatively interpret the results on complex sentence processing under cognitive load as supporting the notion of distinct mechanisms underlying the processing of different sentence structures, in line with the idea of distinct linguistic operations being performed. However, follow-up research is needed to investigate the asserted linguistic operations and their (neural) processing signatures in more detail, possibly with a large-scale study.

## ACKNOWLEDGMENTS

The authors would like to thank all participants for their participation. In addition, the authors would like to thank Jan Michalsky for his help with recording the stimuli, Rebecca Carroll for her help with recording and preparing the stimuli, our students and research assistants (Regina Hert, Laura Peters, Anne Lina Voß, and Charlotte Sielaff) for their help with the data collection, and Gülsen Yanç and Katharina Grote for their support during MRI data acquisition.

## FUNDING INFORMATION

This work was funded by the Deutsche Forschungsgemeinschaft (DFG, German Research Foundation) under Germany’s Excellence Strategy – EXC 2177/1 - Project ID 390895286. This work was supported by the Neuroimaging Unit of the Carl von Ossietzky Universität Oldenburg funded by grants from the German Research Foundation (3T MRI INST 184/152-1 FUGG and MEG INST 184/148-1 FUGG).

## AUTHOR CONTRIBUTIONS

Margreet Vogelzang: Project administration, Conceptualization, Methodology, Formal analysis, Writing. Christiane M. Thiel: Funding acquisition, Conceptualization, Writing. Stephanie Rosemann: Methodology, Data curation, Writing. Jochem W. Rieger: Funding acquisition, Conceptualization, Writing. Esther Ruigendijk: Funding acquisition, Conceptualization, Methodology, Writing.
