# Supplementary material for: Neural Mechanisms Underlying the Processing of Complex Sentences: An fMRI Study
Source: Neurobiol Lang (Camb). 2020 Jun 1;1(2):226–48. doi: 10.1162/nol_a_00011 (PMC10158620; doi:10.1162/nol_a_00011)
Supplement: Supplementary file 1 [file nol-1-2-226-s001.docx]

Supplementary file

for the paper *Neural mechanisms underlying the processing of complex sentences: an fMRI study*

**Activity plots from individual participants**

Activation patterns for the comparisons between sentence types in the single task (complex sentences > SVAO) for all 22 individual participants (p_uncorrected_ < 0.01 on the peak level).

| 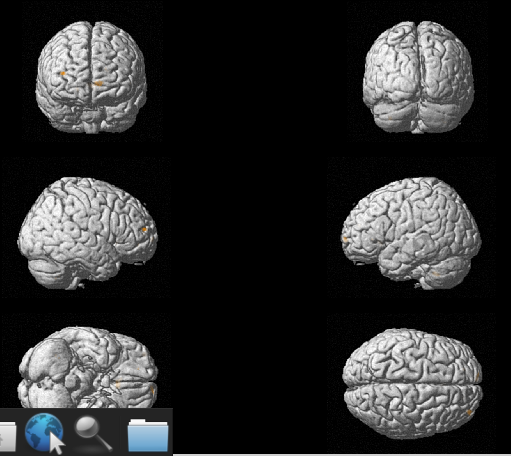 | 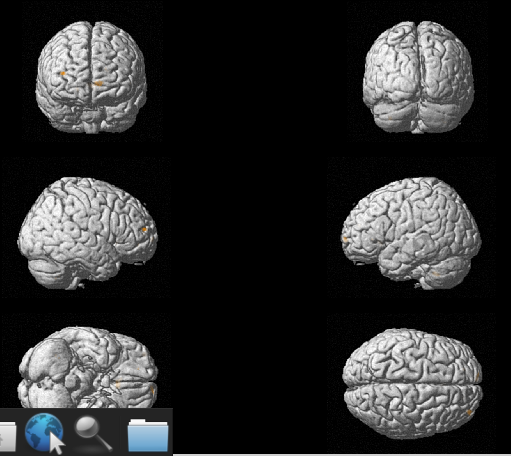 | 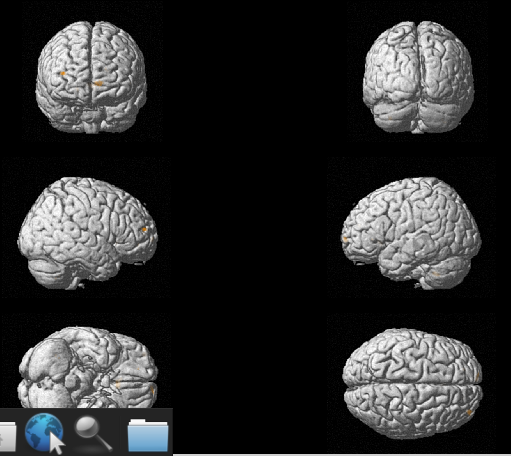 | 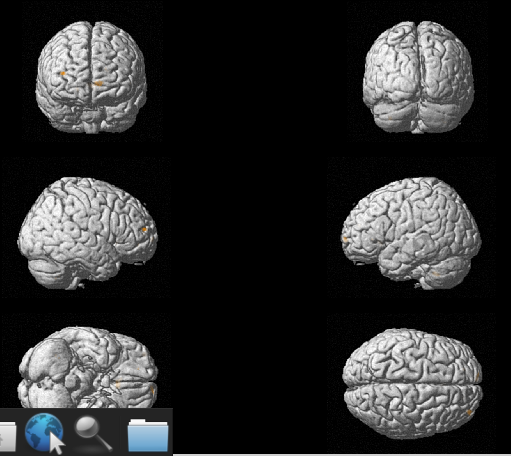 |
| --- | --- | --- | --- |
| 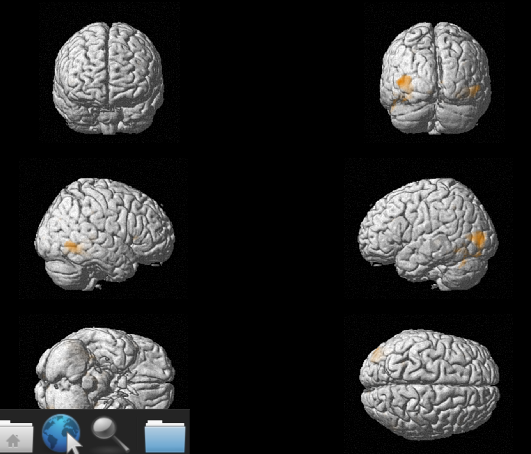 | 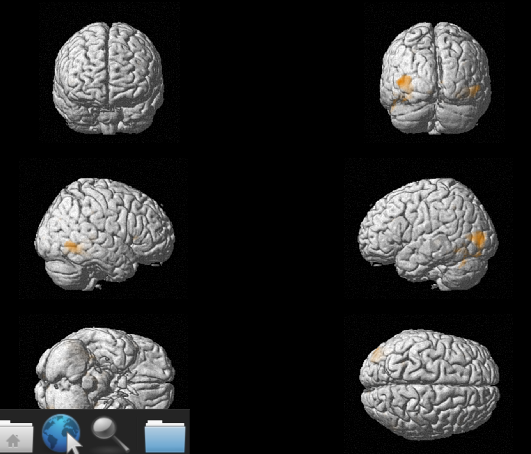 | 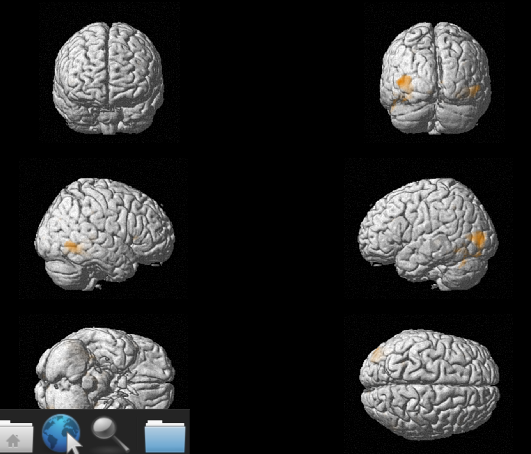 | 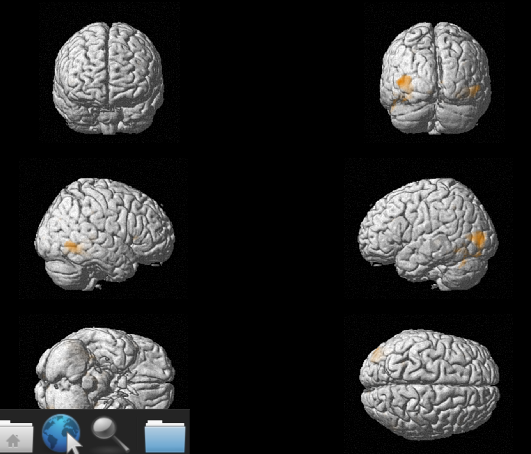 |
| 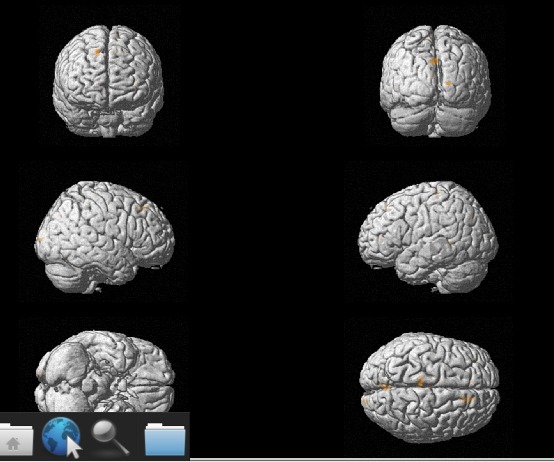 | 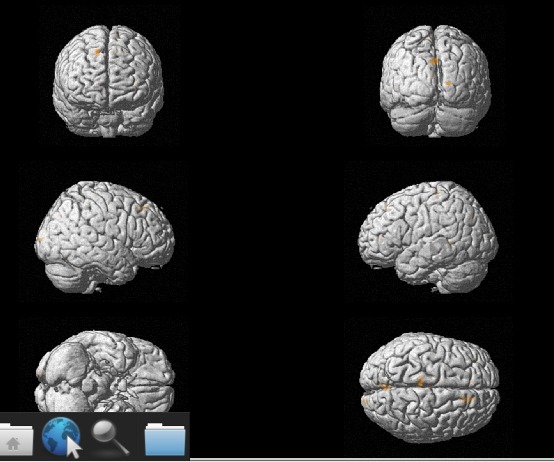 | 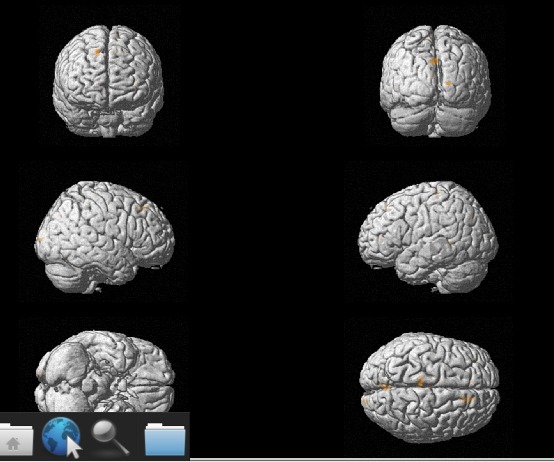 | 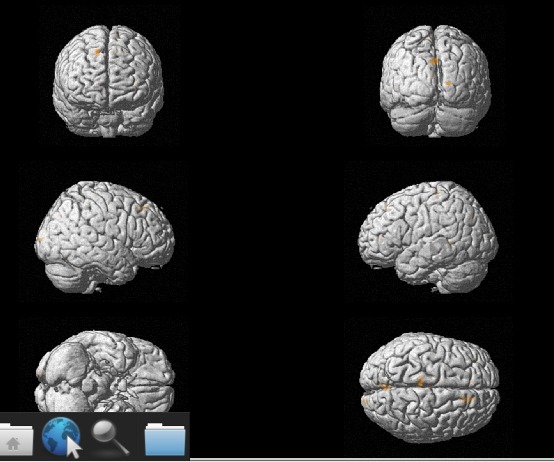 |
| 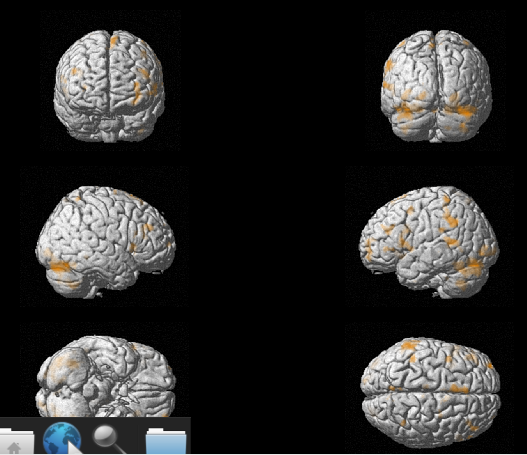 | 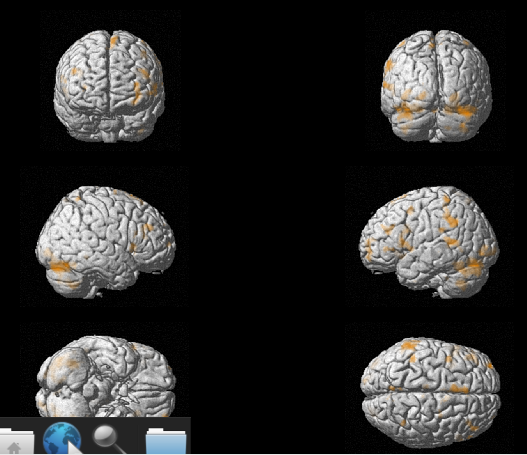 | 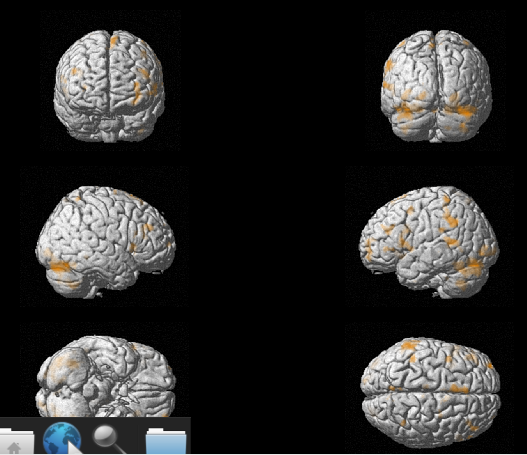 | 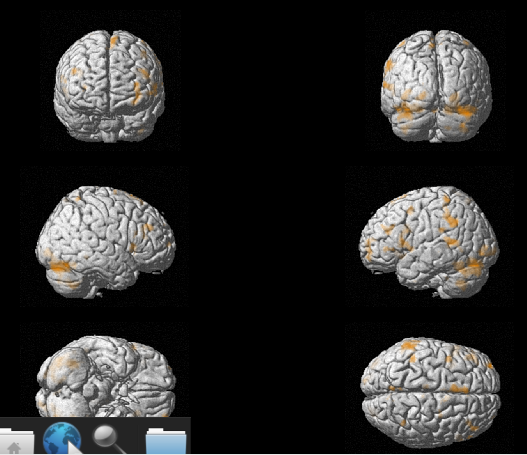 |
| 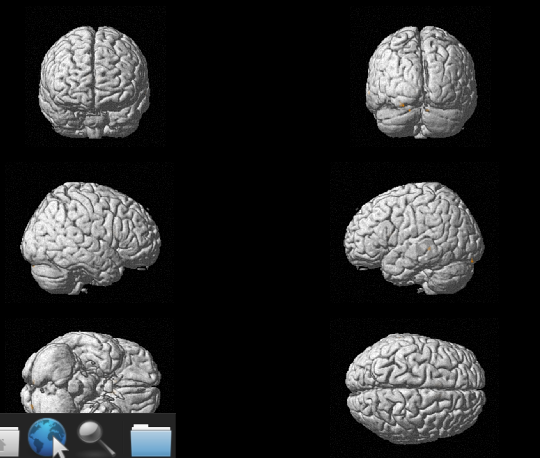 | 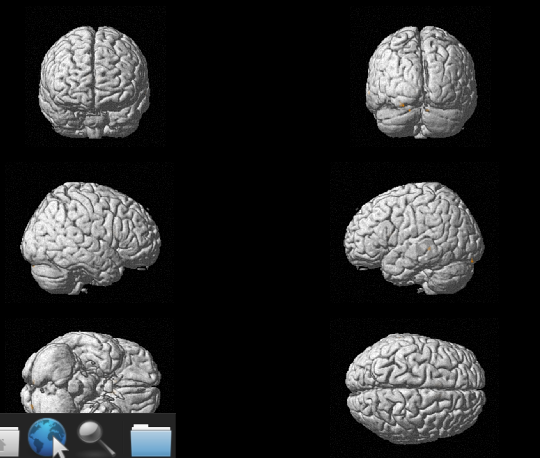 | 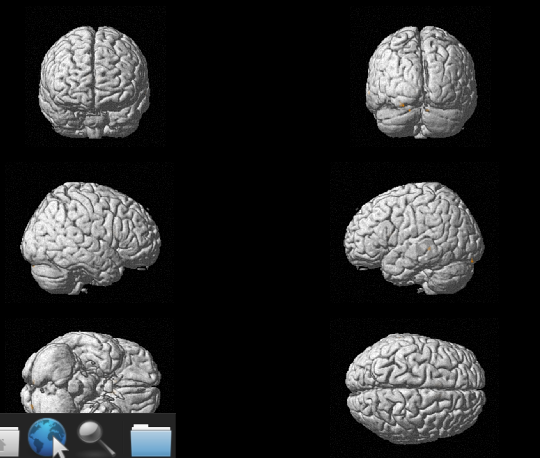 | 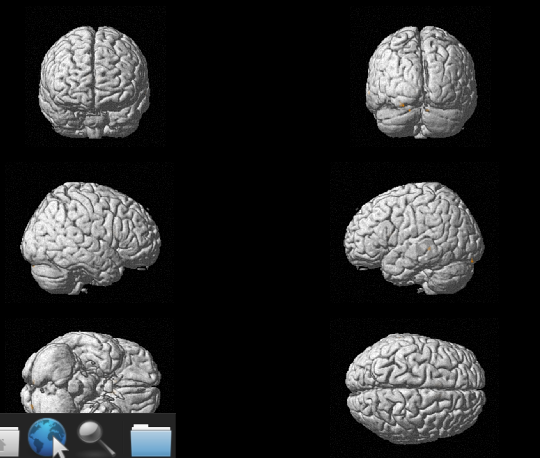 |
| 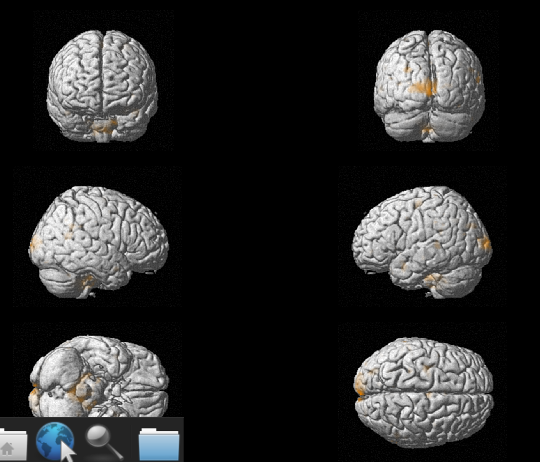 | 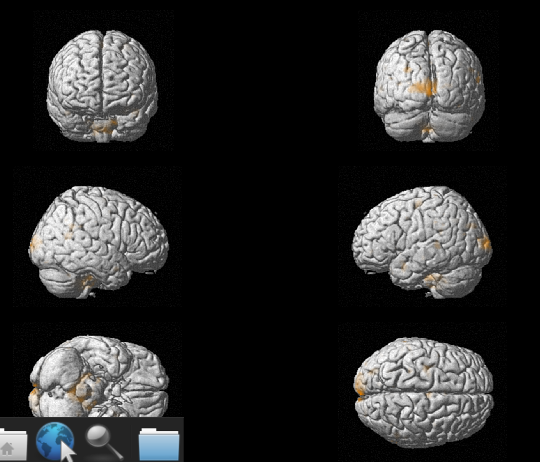 | 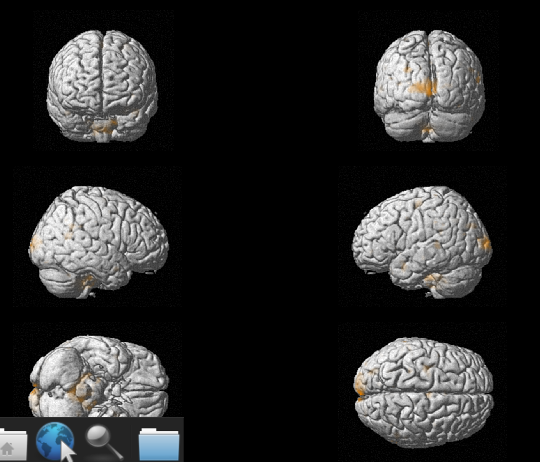 | 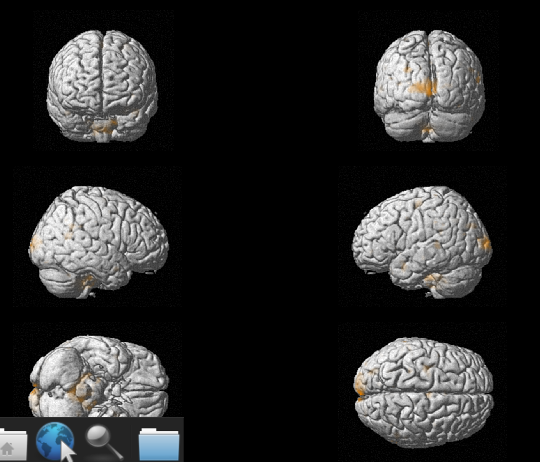 |
| 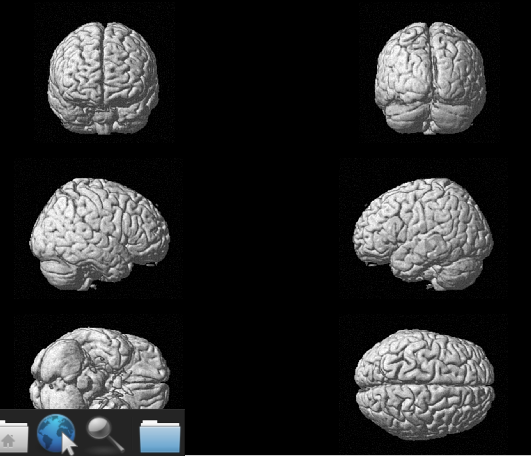 | 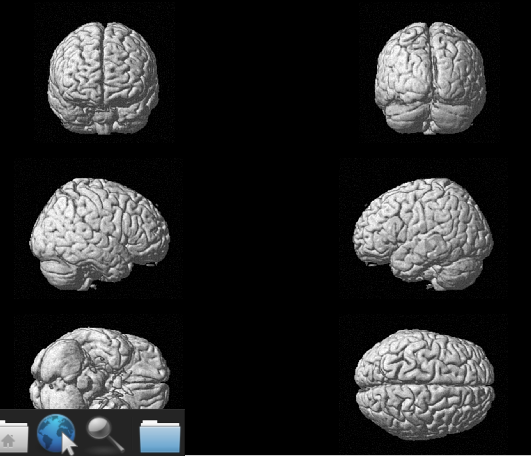 | 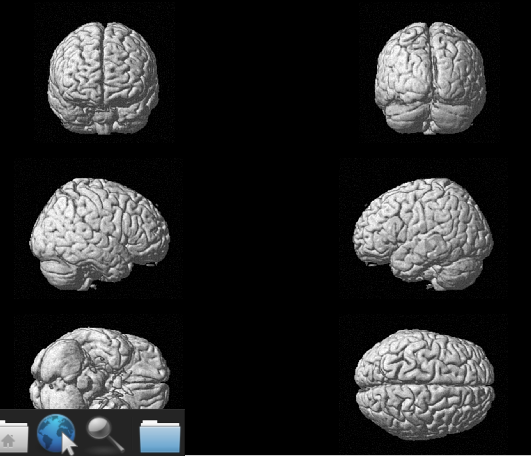 | 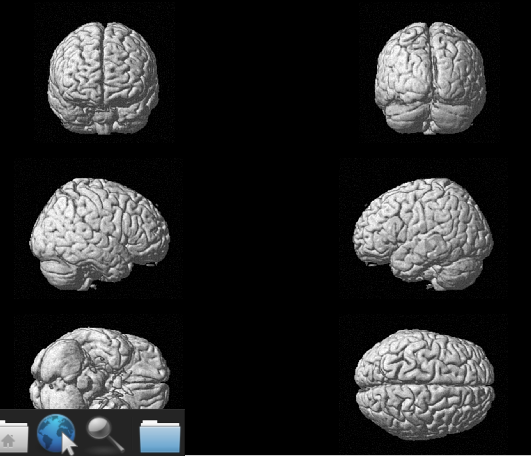 |
| 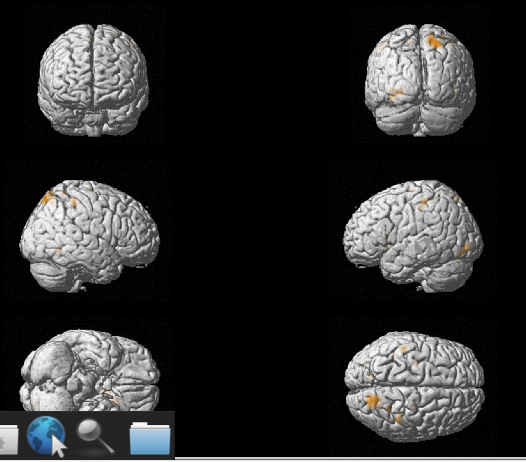 | 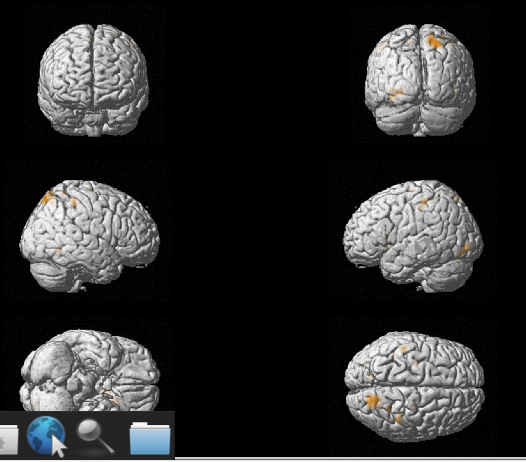 | 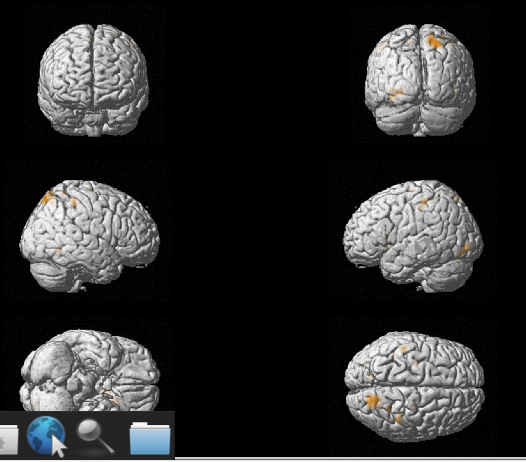 | 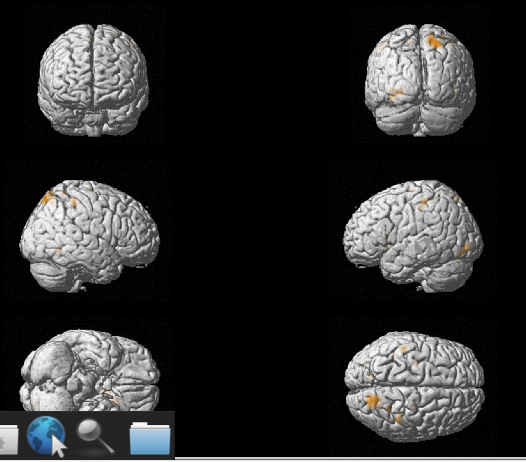 |
| 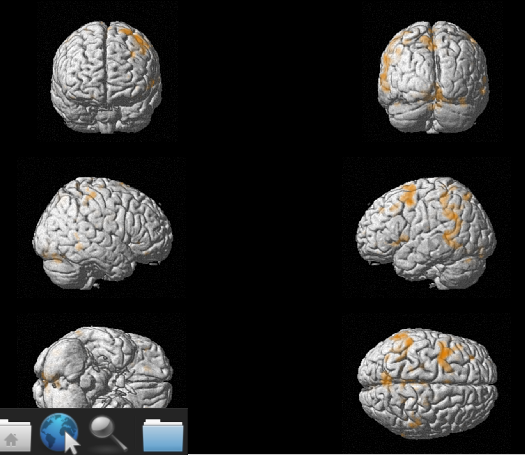 | 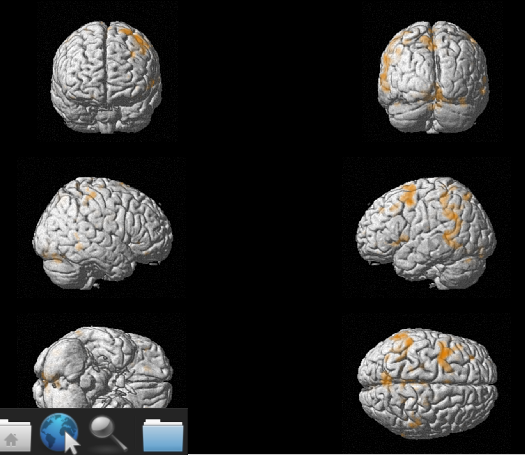 | 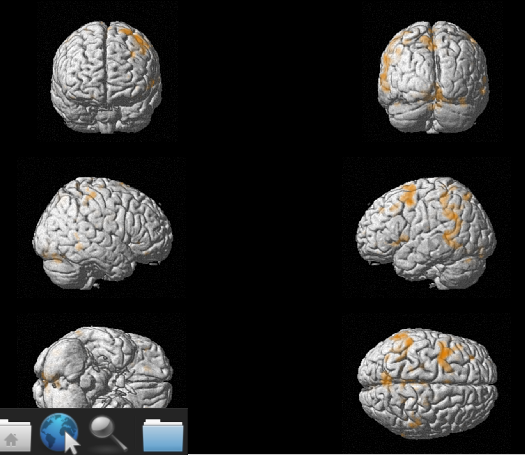 | 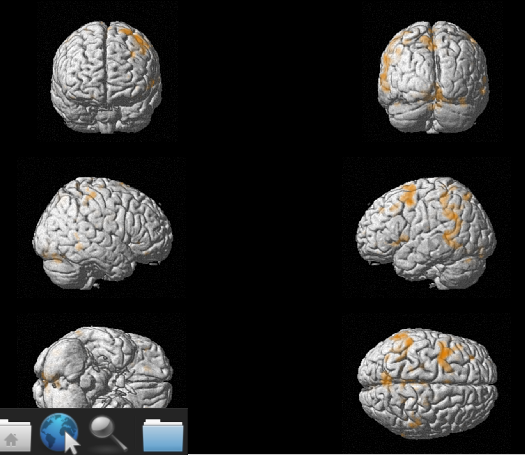 |
| 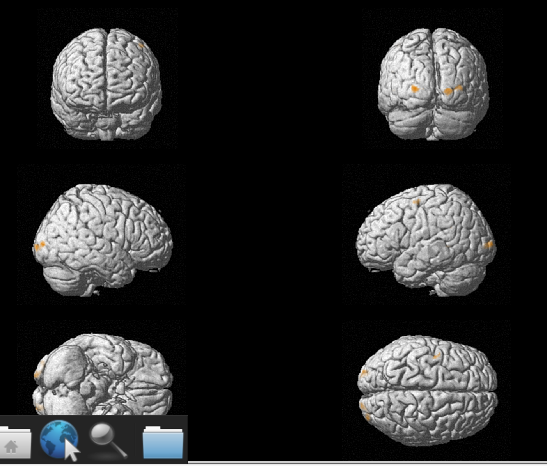 | 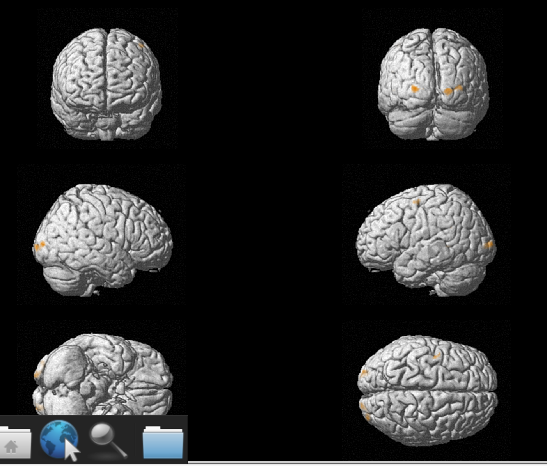 | 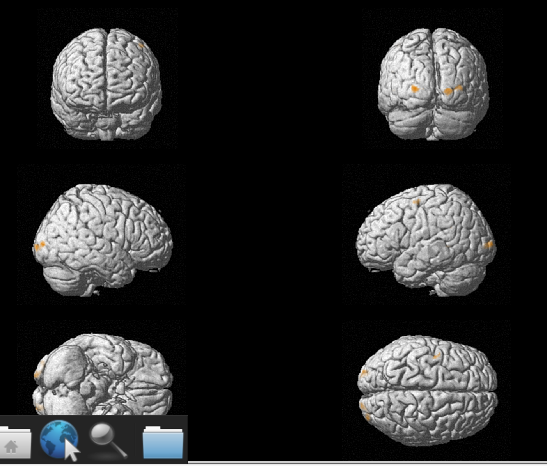 | 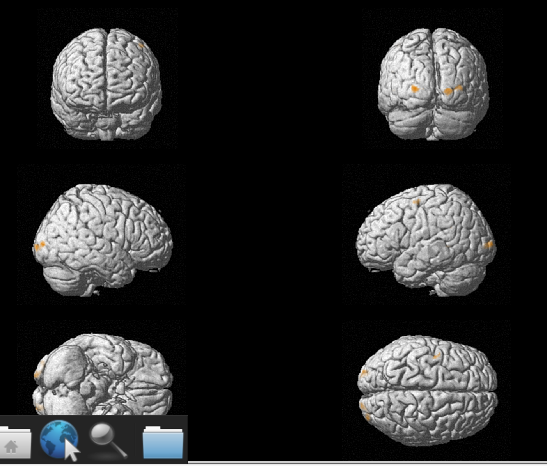 |
| 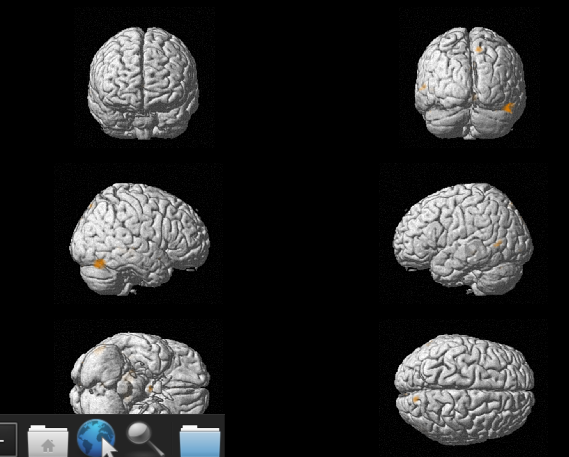 | 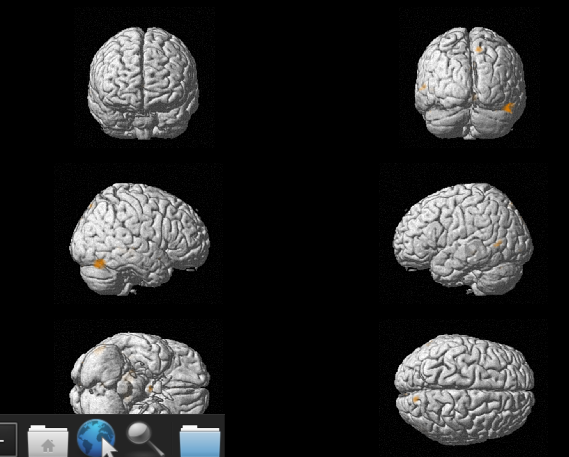 | 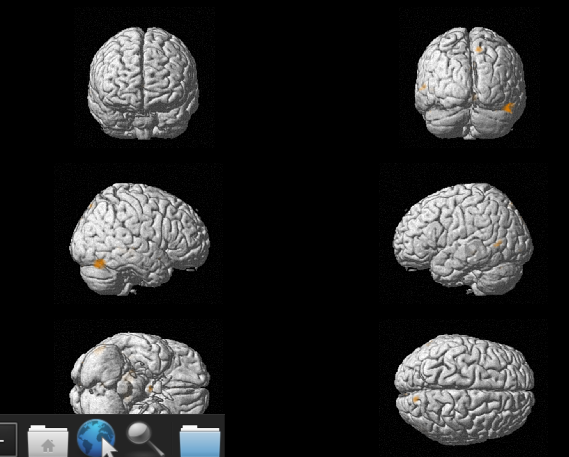 | 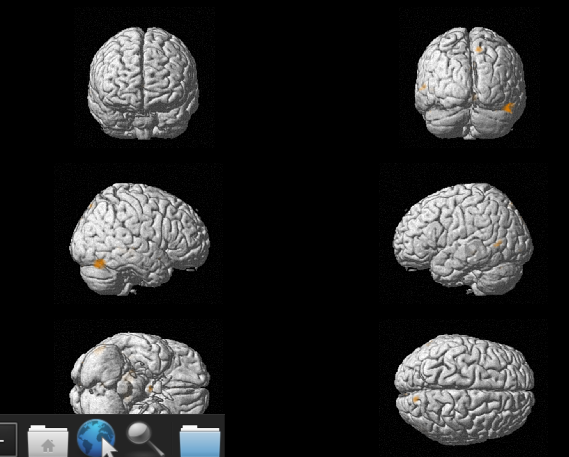 |
| 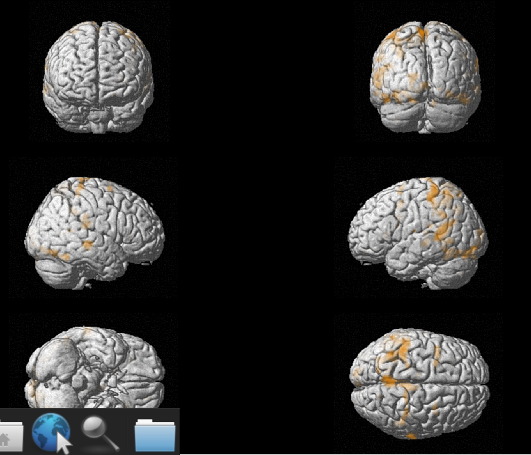 | 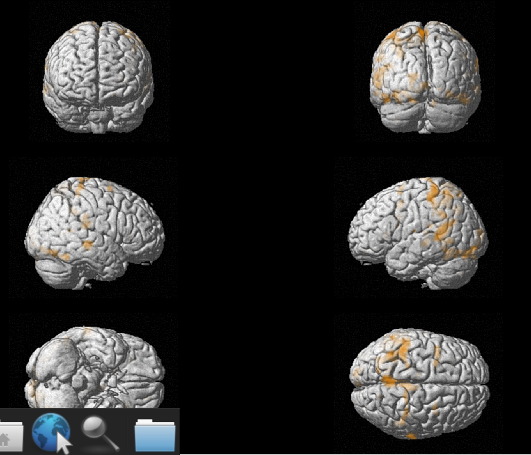 | 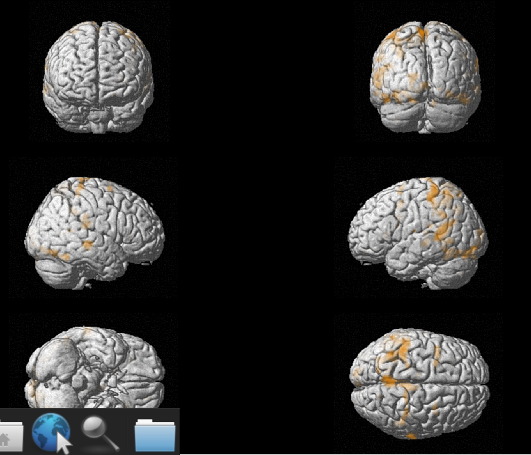 | 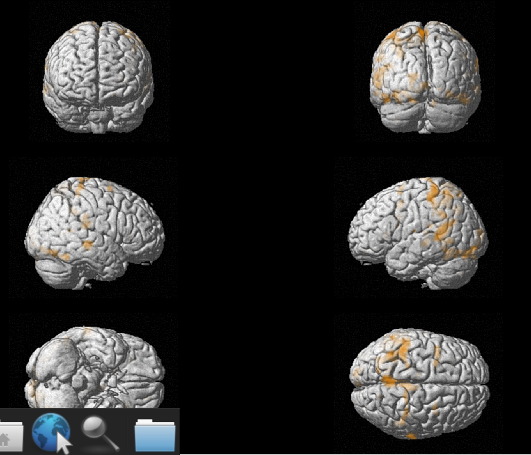 |
| 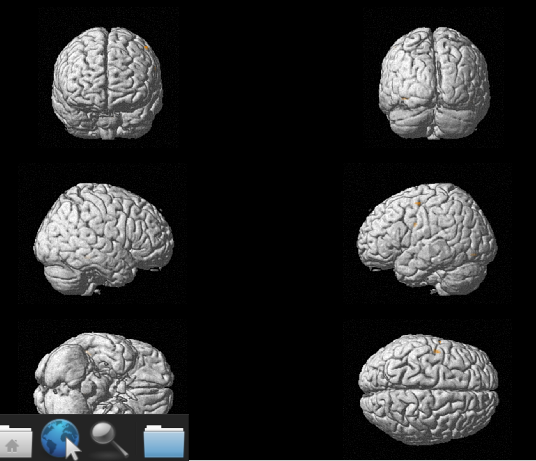 | 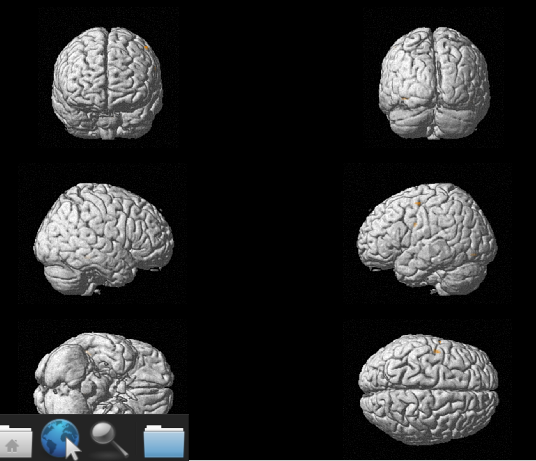 | 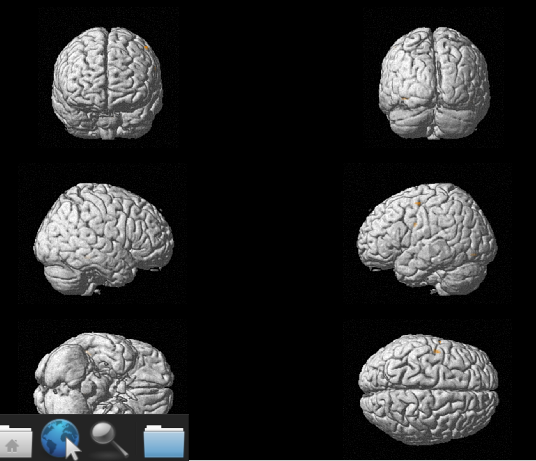 | 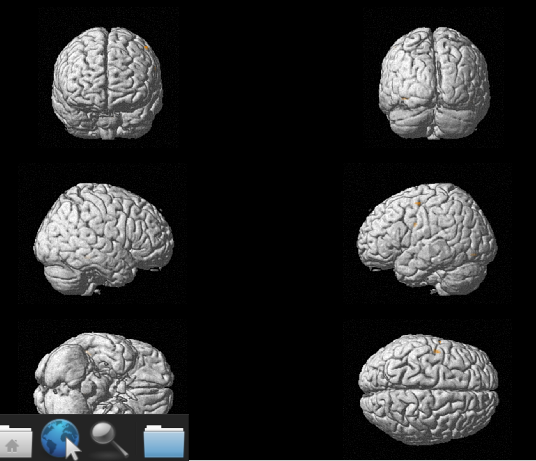 |
| 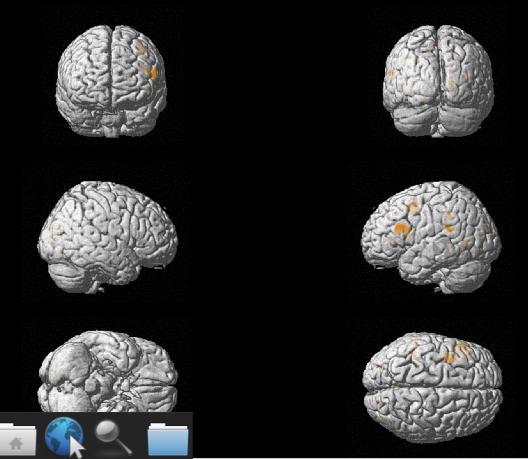 | 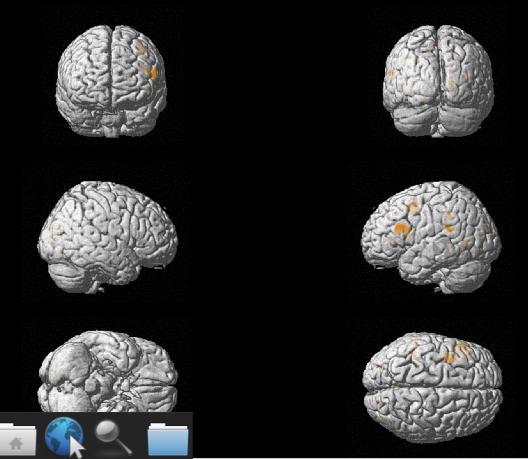 | 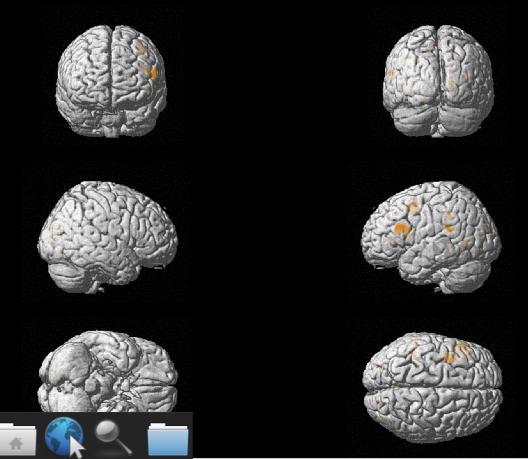 | 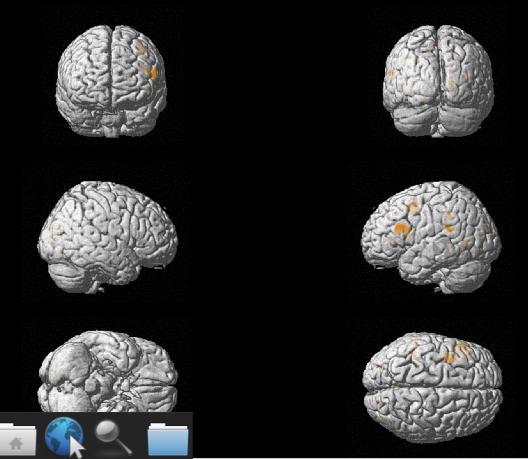 |
| 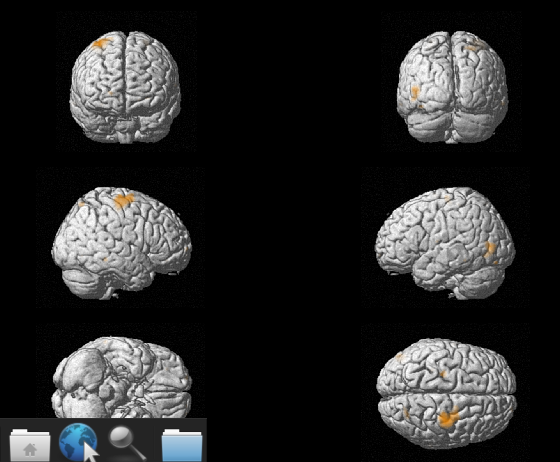 | 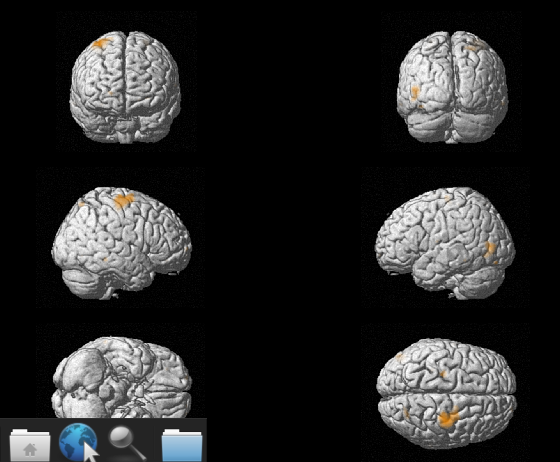 | 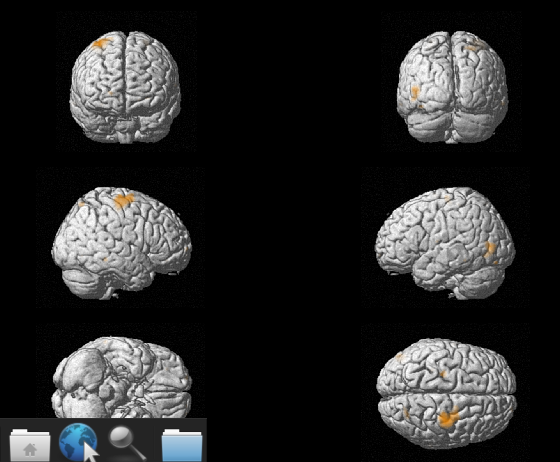 | 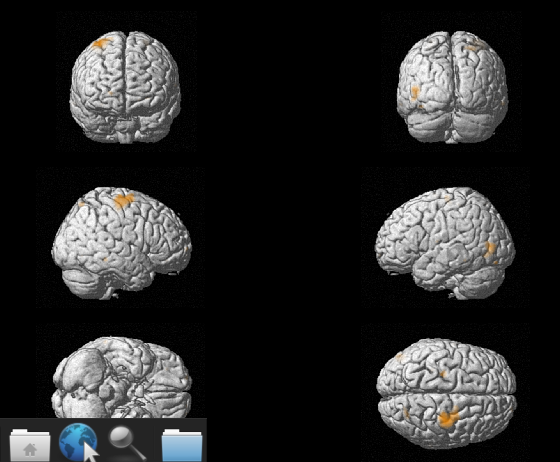 |
| 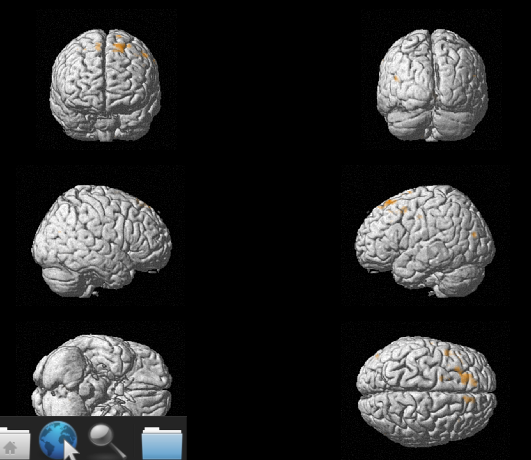 | 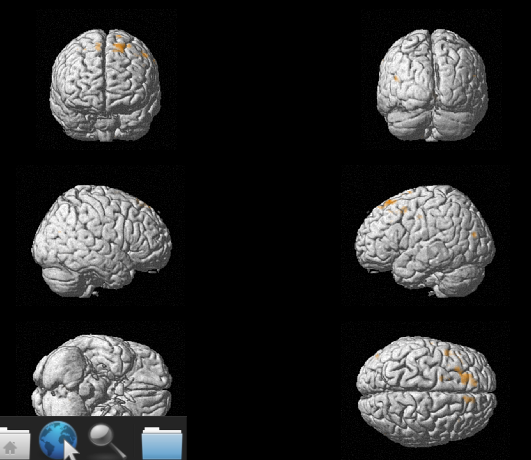 | 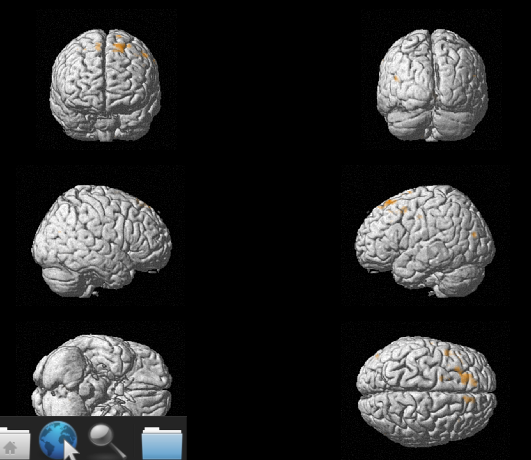 | 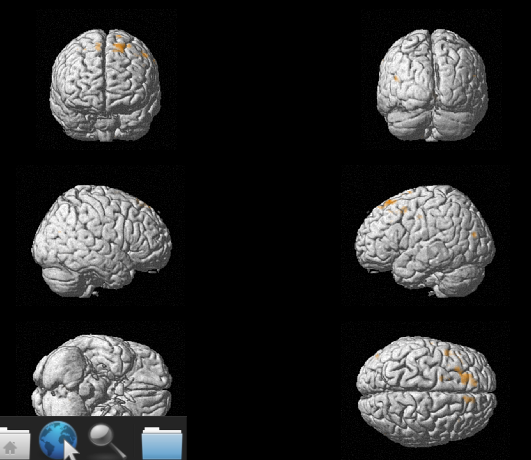 |
| 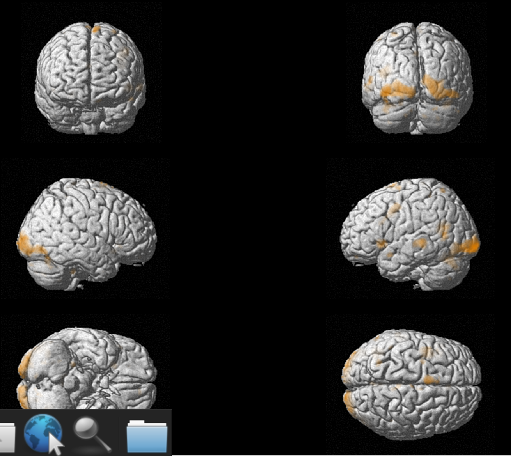 | 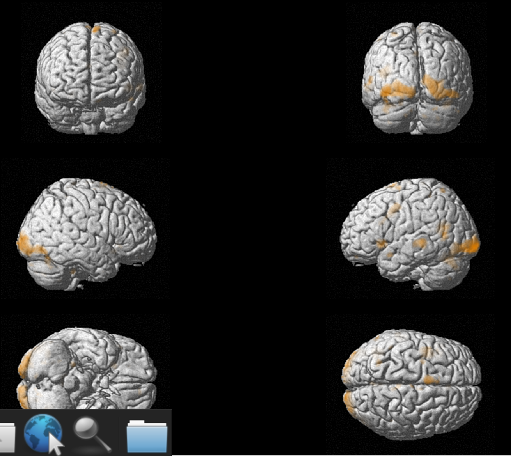 | 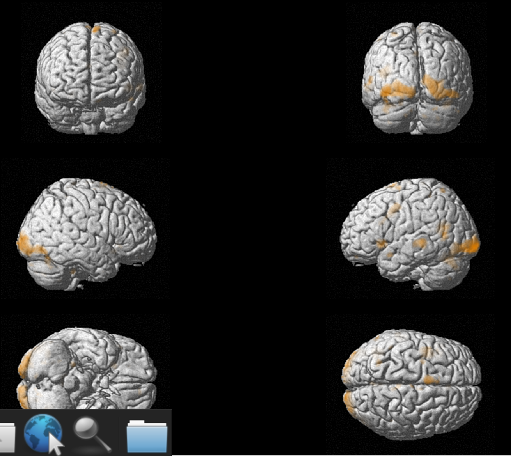 | 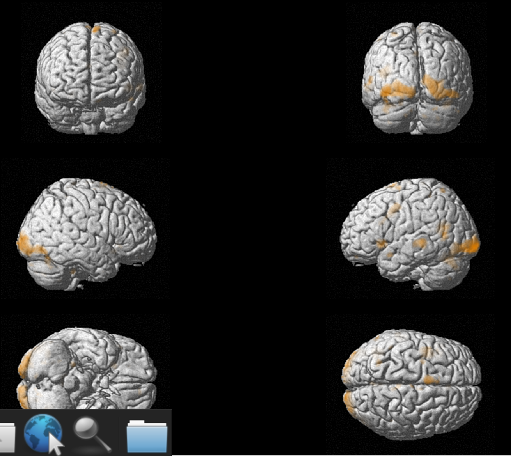 |
| 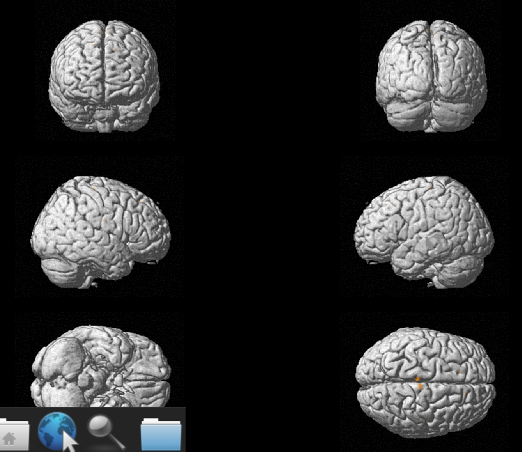 | 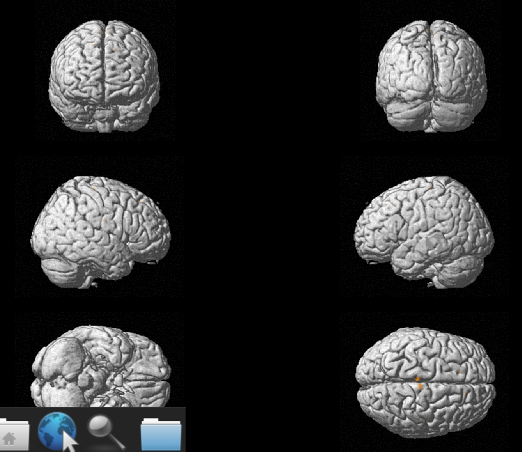 | 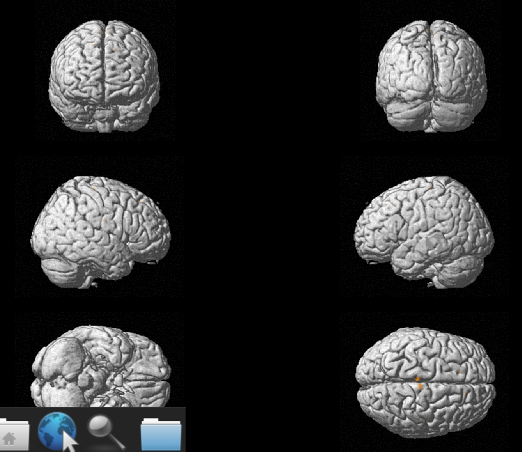 | 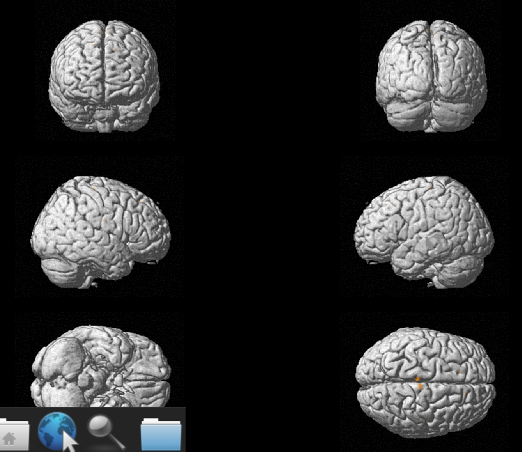 |
| 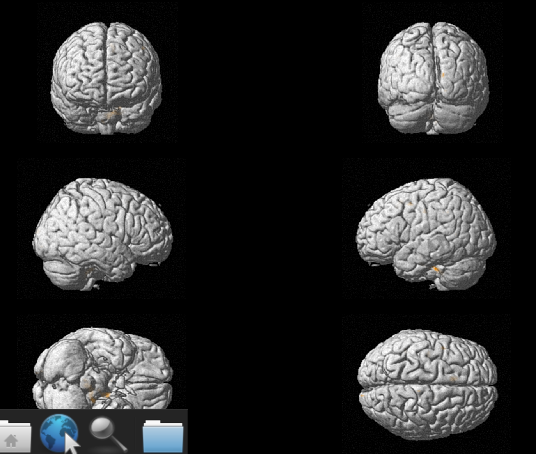 | 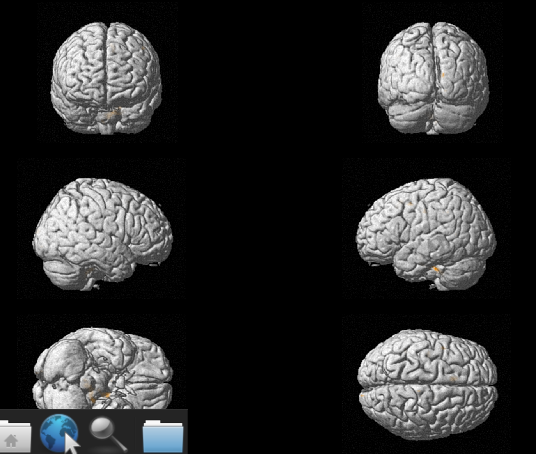 | 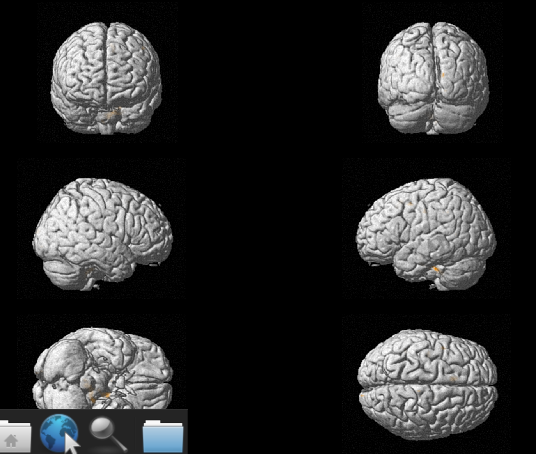 | 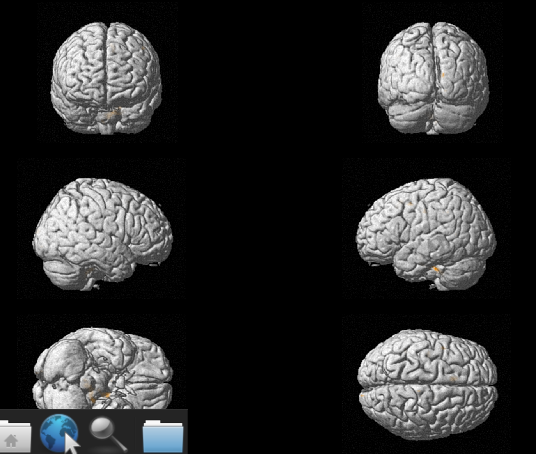 |
| 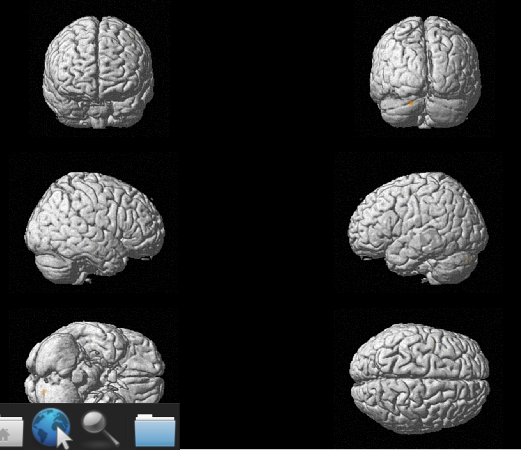 | 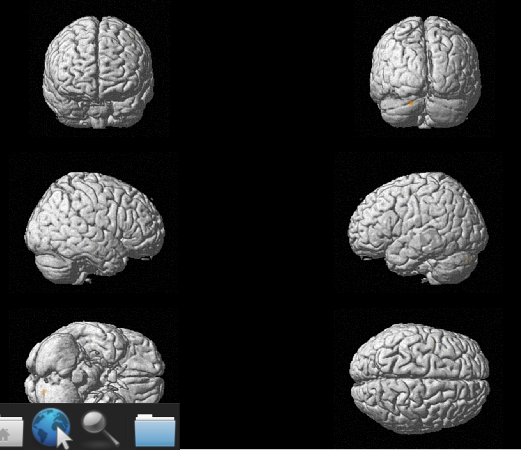 | 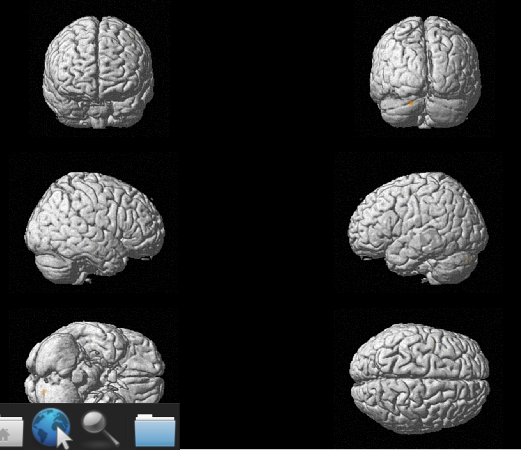 | 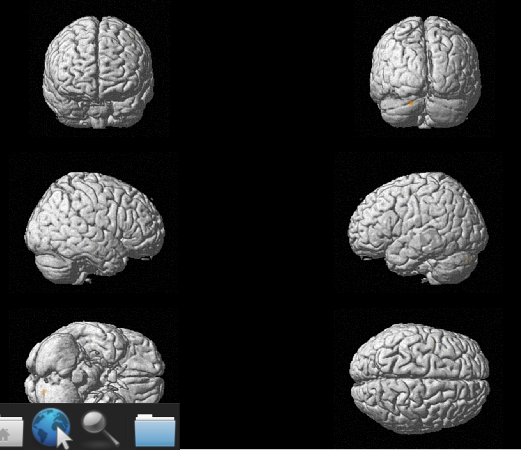 |
| 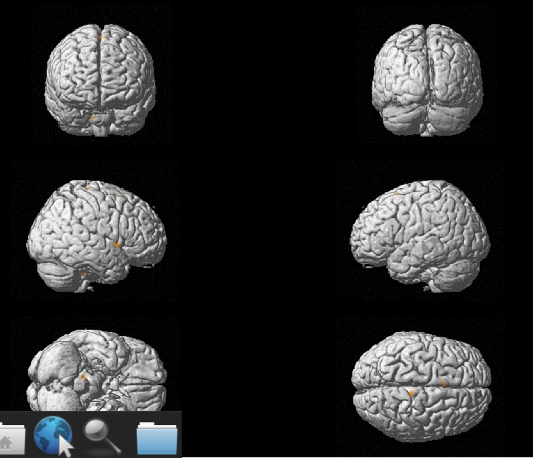 | 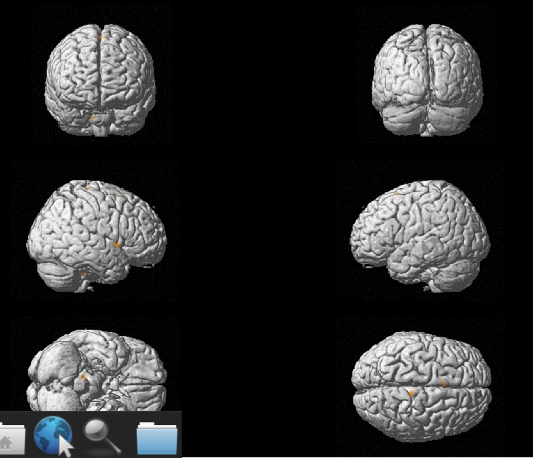 | 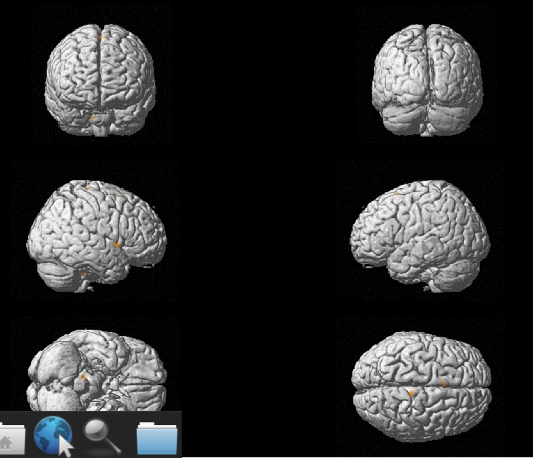 | 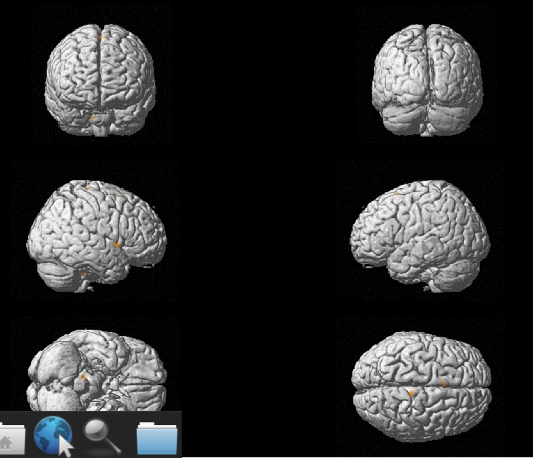 |
| 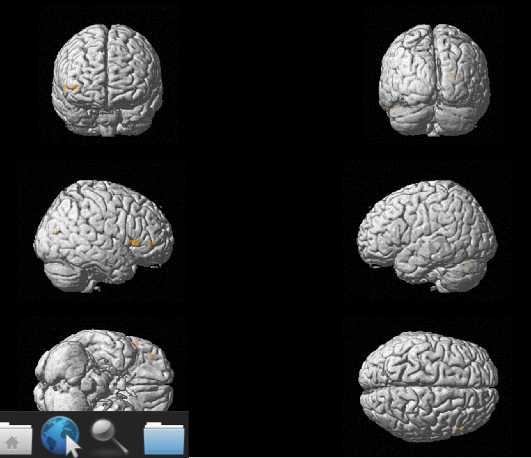 | 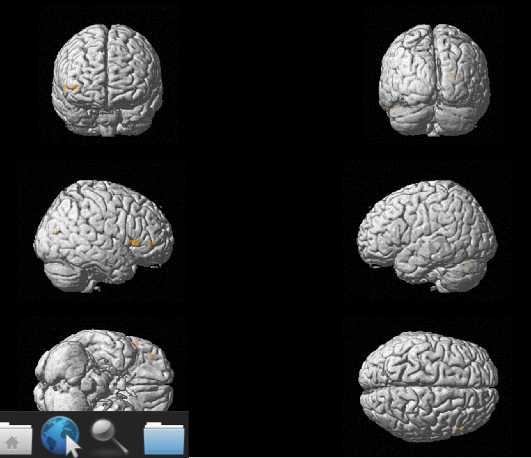 | 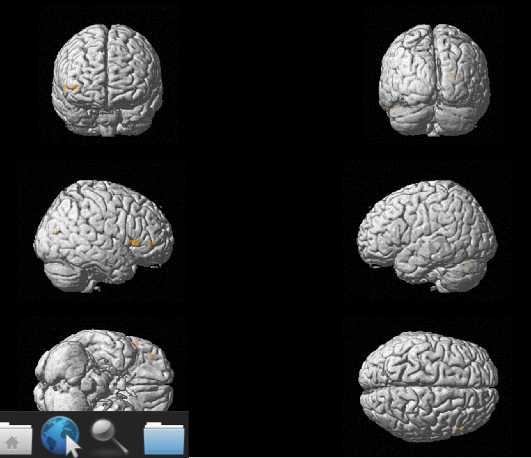 | 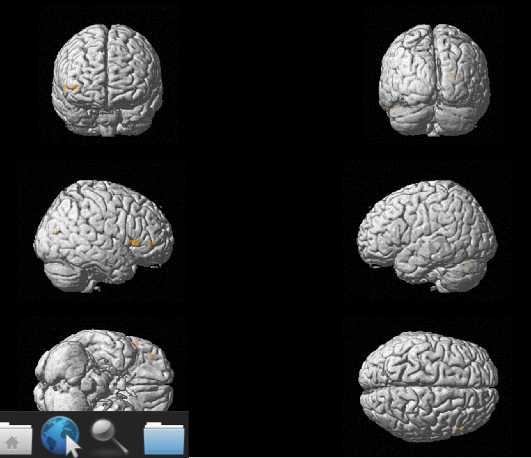 |
